# ACSE: an efficient deep learning model for wheat disease identification

**DOI:** 10.3389/fpls.2026.1838344

**Published:** 2026-06-22

**Authors:** Xiaoyan Yan, Junqi Dong, Longguo Wu, Peng Xu, Zihan Shang, Mohan Liu, Laixiang Xu

**Affiliations:** 1 School of Computer and Artificial Intelligence, Henan University of Urban Construction, Pingdingshan, China; 2 Faculty of Agriculture, Forestry and Ecology, Ningxia University, Yinchuan, China; 3 College of Engineering, Key Laboratory of Modern Agricultural Equipment of Jiangxi Province, Jiangxi Agricultural University, Nanchang, China

**Keywords:** deep learning, dual attention mechanism, robustness, smart agriculture, wheat leaf disease

## Abstract

**Introduction:**

Wheat, as one of the main cereal crops in China, is also one of the most widely planted and high-yielding cereal crops in the world. However, wheat diseases have always been one of the main factors affecting wheat quality and yield. Therefore, accurate diagnosis of wheat diseases is of great significance for wheat production.

**Methods:**

We propose a novel deep learning model based on an improved AlexNet, convolutional block attention module, and squeeze-and-excitation network and call it ACSE. First, the classic AlexNet is optimized to improve its ability to extract complex disease features. Second, a dual channel and spatial combination attention module is designed to extract richer texture features. Finally, a squeeze-and-excitation network is established to enhance the predictive ability and robustness of the model.

**Results:**

The experimental results demonstrate that the proposed model achieves a recognition accuracy of 98.51%. It is superior to other excellent deep learning models such as MobileNetV2, DenseNet, and ShuffleNetV1 in terms of higher recognition precision, stronger adaptive ability, smaller parameter count, lower misjudgment rate, broad universality, and better generalization.

**Discussion:**

The proposed model shows strong potential in the integration of intelligent agricultural systems, which can provide a promising tool for efficient disease diagnosis and help to reduce pesticide abuse and ensure the safety of wheat production.

## 1 Introduction

Smart agriculture is driving the transformation of crop disease monitoring from manual inspection to automated image recognition. Vision-based leaf disease recognition is one of the core technical aspects for achieving precise disease management in the field. Wheat, as an important food crop, is also one of the key pillars of global food security and sustainable agriculture. However, one of the main threats faced by wheat in the production process is various diseases. These diseases seriously affect the health of wheat. To ensure the yield and quality of wheat, the world is continuously strengthening measures for disease prevention and control, including the development of genetically resistant varieties (Wang et al., 2024), improvement of agricultural management technology (Rahman et al., 2025), and establishment of pest and disease monitoring systems. Machine learning and deep learning play a huge role in the field of plant disease recognition.

Traditional methods for identifying plant leaf diseases include symptom observation, microscopic detection, biological assays (An et al., 2023), isolation culture, and inoculation. These approaches are constrained by environmental factors and suffer from high costs, high labor intensity, and strong subjectivity. For instance, symptom observation involves visual assessment of plant discoloration, necrosis, decay, wilting, deformities, and disease manifestations such as mold or powdery mildew. This method heavily relies on human judgment and is prone to misdiagnosis caused by observer bias in large-scale applications. The integration of machine learning has enhanced disease recognition. Bhagat and Kumar (2023) achieved an accuracy of 95.67% by using bag-of-words features with the support vector machine (SVM). Nevertheless, traditional machine learning requires manual feature engineering, and its performance is still limited by training data quality, low computational efficiency, and high resource consumption.

Deep learning models exhibit superior generalization capabilities compared to traditional machine learning approaches. Consequently, deep learning-based algorithms utilize end-to-end feature extraction to enhance robustness in plant disease recognition, integrating lesion localization and disease classification into a unified model. This enables consistent performance across diverse scenarios such as planting fields, while maintaining stability under complex conditions like leaf inclination or blurring. However, these algorithms depend heavily on large-scale, high-quality annotated data, which are costly and difficult to obtain in agricultural contexts. They also demand substantial computational resources and extended training periods, challenging deployment on resource-limited edge devices like field mobiles. Additionally, the black-box nature of deep learning obscures decision-making processes, potentially undermining credibility in expert systems requiring transparent diagnostics. Some studies indicate that deep learning models can overfit specific datasets, resulting in lower generalization ability in real-world complex environments than theoretically anticipated. For example, although Vision Transformer and Swin Transformer have achieved significant success in general benchmarks, their global self-attention mechanism heavily relies on large-scale training data to capture fine-grained disease features. When applied to a limited set of wheat disease data, they are prone to overfitting problems, and due to high computational overhead, they are often difficult to meet the low latency requirements of field-deployable equipment. In addition, its global modeling strategy may inadvertently amplify the background noise in the complex field environment, thus damaging the recognition accuracy. It can be seen that there is still a significant gap between the universality of existing deep learning methods and the specific task of wheat disease recognition. Specifically, there are many kinds of wheat diseases. And the early symptoms of different diseases are highly similar. Field imaging is often accompanied by light changes, leaf overlapping, and soil background interference, which requires the model to make accurate discrimination between fine-grained visual differences and complex backgrounds. At the same time, field real-time detection also puts forward strict constraints on the lightweight level and reasoning efficiency of the model. Therefore, how to build a disease classification model that takes into account recognition accuracy, generalization robustness, and edge deployment efficiency under the condition of limited training samples and computing resources is an urgent problem to be solved. In view of the above challenges, this study proposes the ACSE model.

Based on the above analysis, we propose an integrated deep learning wheat leaf disease recognition model, ACSE. First, we optimize the AlexNet infrastructure through module simplification strategies and channel compression techniques. We replace the 11×11 kernel convolution size in traditional convolutional layers with 8×8, while subtracting one convolutional layer, to reduce the loss of local features during the recognition process. Then, we embed CBAM modules in the convolutional and pooling layers to suppress interference from redundant channels. The CBAM module has a good response effect on leaf occlusion and mixed disease characteristics. Finally, we design the compression and excitation network SENet. The module enhances the network’s ability to capture key information for disease recognition through adaptive feature channel weight allocation and squeeze excitation mechanism. It also compensates for the shortcomings of AlexNet infrastructure in feature screening. It is worth noting that the integration of senet and CBAM is not a simple module superposition. They constitute a progressive collaborative mode in this framework. Senet first focuses on feature selection of channel dimension, adaptively strengthens key disease channels, and suppresses redundant background channels. CBAM then introduced a spatial attention mechanism based on channel attention to accurately locate the spatial position of the lesion area. This complete optimization link from channel filtering to spatial focus has significantly improved the disease identification ability of the model under complex field backgrounds, which are essentially different from the method of using channel attention alone or simply splicing spatial attention. In the experiment, we obtained samples in real-time in different field environments. Therefore, the experimental data has diversity, complexity, and uncertainty. This greatly restores the real planting situation and improves the credibility of the experimental results. At the same time, We identified multiple diseases on wheat leaves and achieved a classification accuracy of 98.51%. The main contributions of this paper are as follows:

1. We reconstruct the classical AlexNet network layer, divide and disassemble the large core convolution of the traditional convolution layer 11×11, and remove some redundant structures, such as too many ReLU layers. So as to achieve the simultaneous improvement of lightweight deployment and perception performance of the model while reducing the high computational cost.
2. We propose a novel model component integration strategy that hierarchically fuses the CBAM attention mechanism (capturing dual channel-spatial dimensional features) and SENet (intensely focusing on channel-wise features) via convolutional layers to build a cross-layer connected multi-stage feature structure. This design reinforces discriminative feature mining, enhances the model’s feature extraction and expression capabilities, and concurrently improves recognition accuracy.
3. We propose a cross-sample statistics regularization method that constrains the mean and variance of inputs to each layer within a stable interval, effectively suppressing distribution fluctuations in deep networks, mitigating gradient calculation biases caused by distribution shifts, and improving training stability and generalization performance, enabling the model to perform well even with only a small amount of data.
4. In order to ensure the authenticity and accuracy of the experiment, the proposed method is compared with other classic models. In the same environment, the self-built and public wheat data sets are analyzed according to a number of parameter indicators. Many results show that the method proposed by us is effective.

Overall, we present a task-oriented optimization strategy for the AlexNet framework that accomplishes bidirectional optimization of channel feature screening and channel spatial feature collaborative augmentation, rather than merely stacking attention modules. We have made improvements to AlexNet’s original 5-layer convolution by embedding attention mechanisms between key convolution layers and pooling layers to enhance the model’s ability to identify wheat leaf disease features. We establish the SENet to focus on feature screening at the channel dimension, adaptively strengthening critical disease channels and suppressing redundant background channels through a squeeze excitation mechanism. The CBAM module also includes a spatial attention mechanism based on channel attention, which accurately locates the spatial position information of illness areas and improves the ability to detect subtle disease spots. These two modules form a progressive collaborative mode of channel filtering and spatial focusing, constructing a complete chain of feature optimization. It is fundamentally different from methods that use channel attention alone or simply concatenate spatial attention. Through the complementary collaboration of dual mechanisms, the model performs better in disease recognition tasks in complex backgrounds. Thanks to this collaborative design, the proposed scheme achieves a significant improvement in recognition accuracy while maintaining the lightweight structure. Its small number of parameters and low computational overhead make it a promising candidate for the deployment of resource-constrained edge devices in future smart agriculture scenarios.

The remaining content of this article is organized as follows: Section 2 summarizes the literature review, clarifies the research background and current status of plant disease identification, and provides the theoretical basis for the research project. Section 3 proposes the construction method and mathematical principles of the new model ACSE. Section 4 introduces the experimental results and analysis. Section 5 presents the findings discussion. Finally, Section 6 summarizes and looks forward to this experiment.

## 2 Literature reviews

The evolution of crop disease identification technology is closely linked to breakthroughs in information science. Wheat, as a major global cereal crop, has made the intelligent detection of its diseases a persistent research focus in the field of agricultural artificial intelligence. Against the backdrop of continued global climate change and intensive planting patterns, the frequency and transmission rate of wheat diseases have significantly increased. Traditional identification methods that rely on manual experience are no longer able to meet the urgent needs of modern agriculture for precise and efficient disease prevention and control. In recent years, the rapid development of machine learning and deep learning technologies has offered novel solutions for wheat disease identification. Related research has gradually expanded from initial image classification to multi-modal data fusion, lightweight model deployment, and cross-scene collaborative applications, gradually building a more systematic technical framework.

Machine learning (ML) methods laid the foundation for automated wheat disease recognition by learning mappings between handcrafted visual features and disease categories, preceding deep learning. They convert raw images into discriminative feature vectors via operators tailored to disease morphology: color features are quantified by chromatic space mean and variance to distinguish healthy from infected tissues; texture patterns particularly diagnostic of stripe rust are captured using gray-level co-occurrence matrices (GLCM) and local binary patterns (LBP); shape descriptors extract geometric parameters (area, perimeter, circularity) via edge and contour analysis. The classifiers usually use SVM, random forest (RF), and KNN, with SVM outperforming in small-sample scenarios via optimal hyperplane construction.

Machine learning has established a foundation for automated wheat disease detection but encounters significant practical limitations. First, these methods heavily rely on expert prior knowledge for manual feature design. The feature extraction process is time-consuming and labor-intensive, and the resulting representations are limited, making it difficult to comprehensively detect and localize disease features in complex environments. Second, the separation of feature extraction and classification impedes end-to-end optimization, constraining accuracy and robustness. Additionally, ML models exhibit low sensitivity to subtle lesions, complex infections, and early disease manifestations, causing missed detections, alongside poor adaptability to diverse regional, varietal, and cultivation conditions, resulting in weak transferability and frequent redesign needs for new environments. Consequently, ML’s inherent bottlenecks have shifted research focus toward deep learning, which excels in automatic discriminative feature learning and end-to-end optimization advantages.

Convolutional neural networks (CNNs) are widely used in wheat disease recognition, typically comprising convolutional, pooling, and fully connected layers. Enhanced network depth and width improve representational learning; for instance, VGG16 achieves over 90% accuracy in wheat stripe rust recognition by capturing fine lesion textures and color distributions. To mitigate gradient vanishing in deep networks, ResNet employs cross-layer identity mapping, enabling hundred-layer depths and superior complex feature modeling, as evidenced in wheat disease classification. Despite high accuracy in diverse tasks, large-scale parameters often cause overfitting and limit generalization with small wheat disease datasets. In addition, the original convolution design has insufficient ability to capture fine-grained disease features in the complex background of the field, which further limits the generalisation performance of these classical architectures in the task of wheat disease recognition. This limitation also constitutes an important motivation for subsequent research to evolve towards lightweight.

Recent years have seen successful adaptation of Transformer architectures from natural language processing to computer vision, revolutionizing wheat disease identification. Unlike CNNs that capture local features, Transformers use self-attention to model long-range dependencies across image patches, enabling holistic discrimination between diseased and healthy tissue. The Vision Transformer (ViT) achieves robust performance by treating images as patch sequences; Rezaei et al. (2024) reported 90.12% accuracy on PlantDoc using a ViT-based PMF+FA method. The Swin Transformer, with hierarchical design and sliding-window attention, reduces computational overhead while preserving global context; Yang et al. (2023) demonstrated 99.00% classification and 88.73% severity assessment accuracy with their three-branch Swin Transformer classifier (TSTC), outperforming conventional CNNs. Additionally, large-scale pretrained visual models excel in few-shot agricultural learning: Zhang et al. (2024) introduced the Wheat Disease Language Model (WDLM), integrating an enhanced Segment Anything Model with a fine-tuned large language model and chain-of-reasoning to achieve superior accuracy in complex fields and enable mobile real-time diagnosis. Although the above methods have achieved excellent results in benchmark testing, their excellent performance is highly dependent on large-scale training data and intensive computing resources. In the small agricultural data sets and the field environment with limited resources, these models are prone to serious overfitting problems, and their huge parameters also cause the delay of real-time reasoning that is difficult to ignore, which brings significant challenges to actual deployment.

Recent advances in lightweight deep learning have significantly enhanced field-deployable wheat disease identification. EfficientNet models, optimized via composite scaling of depth, width, and resolution, deliver superior accuracy-efficiency trade-offs under constrained computational budgets, with EfficientNet-B0 enabling real-time inference on UAVs and EfficientNet-B2 achieving top performance in comparative studies. Hybrid frameworks such as CDIP-ChatGLM3 integrate vision transformers with fine-tuned large language models to simultaneously diagnose diseases and generate actionable prescriptions. Although these methods reduce the number of parameters and calculations, the existing lightweight models often sacrifice the fine perception ability of wheat leaf microlesions and complex texture in the process of pursuing efficiency, resulting in a significant decline in the recognition accuracy in the actual deployment in the field. At the same time, their generalisation robustness in cross-environment and cross-species scenarios is still not ideal, which makes it a key problem to be solved urgently to maintain the diagnosis accuracy without damaging the reliability under the condition of resource constraints. Most of the above lightweight models are designed for general visual tasks, and their internal feature extraction structure may not be well adapted to fine-grained recognition requirements such as the variable scale of wheat disease spots and fuzzy boundaries. In a specific agricultural scenario, the task-oriented deep optimisation of the proven classic architecture is often more practical than directly using the novel backbone network with a complex structure.

Data scarcity, category imbalance, and poor image quality remain critical bottlenecks in wheat disease recognition. To address these, few-shot learning (FSL) enables effective recognition with minimal labeled samples, as shown by Rezaei et al. (2025) who achieved 91.80% and 97.93% accuracy on barley and cassava using a Swin-B transformer framework. Traditional augmentations, rotation, cropping, and color jitter effectively model field variability, while oversampling techniques adapted from corn phenology studies (Feng et al., 2025) mitigate class imbalance in powdery mildew datasets. Semi-supervised and self-supervised methods like CLA (Zhao et al., 2024) reduce annotation dependency, achieving 90.52% accuracy on leaf diseases and enhancing generalization with UAV-collected imagery, forming a robust pipeline advancing real-world wheat disease diagnosis under resource constraints.

The integration of Internet of Things (IoT) technologies has enabled scalable, real-time wheat disease monitoring through synergistic deployment of UAVs, ground sensor networks, and edge computing. UAVs equipped with multispectral sensors acquire high-resolution imagery (10 cm), enabling batch disease detection via deep learning; Zeng et al. (2024) achieved 98.44% accuracy in powdery mildew identification using a multi-scale selective attention CNN, a framework adaptable to wheat fields for precision spraying. Concurrently, ground sensors monitor environmental variables, temperature, humidity, soil moisture, and spectral reflectance to assess disease risk dynamically. Diachenko et al. (2025) demonstrated an 82% prediction accuracy for powdery mildew onset using weather-based machine learning models, validating their utility in early warning. Fusion of UAV-derived spectral data with *in-situ* sensor inputs significantly enhances diagnostic reliability, particularly for scab prediction, where soil moisture correlates strongly with infection probability. This multi-modal IoT architecture reduces reliance on manual scouting, supports data-driven decision-making, and establishes a robust foundation for scalable smart agriculture systems. The above multimodal collaborative architecture integrates UAV remote sensing, ground sensor networks, and edge computing and outlines a complete technical closed loop from data acquisition to decision execution for smart agriculture. However, the reliable operation of the closed-loop is still based on the perception accuracy of the underlying visual recognition unit under complex field conditions. This study focuses on the image classification task in this basic link, aiming to provide accurate disease recognition ability for the upper monitoring system.

## 3 Proposed methods

### 3.1 Optimized AlexNet

In wheat leaf disease recognition, models must capture both local lesion features (e.g., powdery mildew’s white spores, downy mildew’s yellow polygonal lesions) and global characteristics like leaf morphology and lesion distribution density. While AlexNet’s deep convolutional architecture provides foundational feature extraction capabilities, its original design exhibits limitations in field applications. The 11×11 large-kernel convolutions cause feature dilution in small wheat leaves with blurred lesion boundaries, leading to suboptimal feature localization. Furthermore, the baseline channel configuration and layer stacking inadequately exploit disease-specific color channel variances under natural conditions. The parameter-heavy fully-connected layers also incur high computational overhead, impeding real-time deployment on portable field devices and compromising practical utility in disease surveillance systems.

Although new backbone networks with superior performance, such as EfficientNet and Swing Transformer, have emerged in recent years, AlexNet is still used as the infrastructure in this study, which is rooted in the particularity of the wheat disease identification task. Firstly, the large-scale shallow convolution kernel adopted by AlexNet has natural advantages in capturing the underlying visual features such as leaf surface texture, edges, and local colour anomalies, which are exactly the important basis for early identification of wheat disease spots. Secondly, compared with complex structures such as composite scaling or self-attention, AlexNet’s module composition is simple and transparent, and the parameter space is controllable. Under the constraints of limited samples in the agricultural scene, AlexNet can effectively reduce the risk of overfitting and provide a clear optimisation path for the subsequent directional lightweight transformation. Finally, the balance between efficiency and accuracy achieved by task-level customization of mature architecture is often better than directly transplanting novel models that have not been fully verified in agricultural vision tasks.

The model selected for this study requires robust feature extraction capabilities and compatibility with large-scale dataset training. Considering the sample size and intrinsic characteristics of the wheat leaf disease dataset, we optimized the network architecture of the classic AlexNet model. In terms of convolution layers, the 11×11 convolution kernel used by the original AlexNet is easy to introduce too much background information into the receptive field of wheat leaf disease spots, resulting in local feature dilution. In order to determine a more appropriate convolution kernel size, we conducted comparative tests on five configurations, namely, 3×3, 5×5, 7×7, 8×8, and the original 11×11, while keeping the rest of the network structure unchanged. The results are shown in **Table 1**.

**Table 1:** Comparison of classification performance under different convolution kernel sizes.

Kernel size	Parameters (M)	FLOPs (G)	Accuracy (%)
3×3	2.16	0.28	94.72
5×5	2.37	0.41	95.38
7×7	2.68	0.59	96.15
8×8	2.89	0.68	96.83
11×11	3.52	0.95	95.46

**Figure 1:**
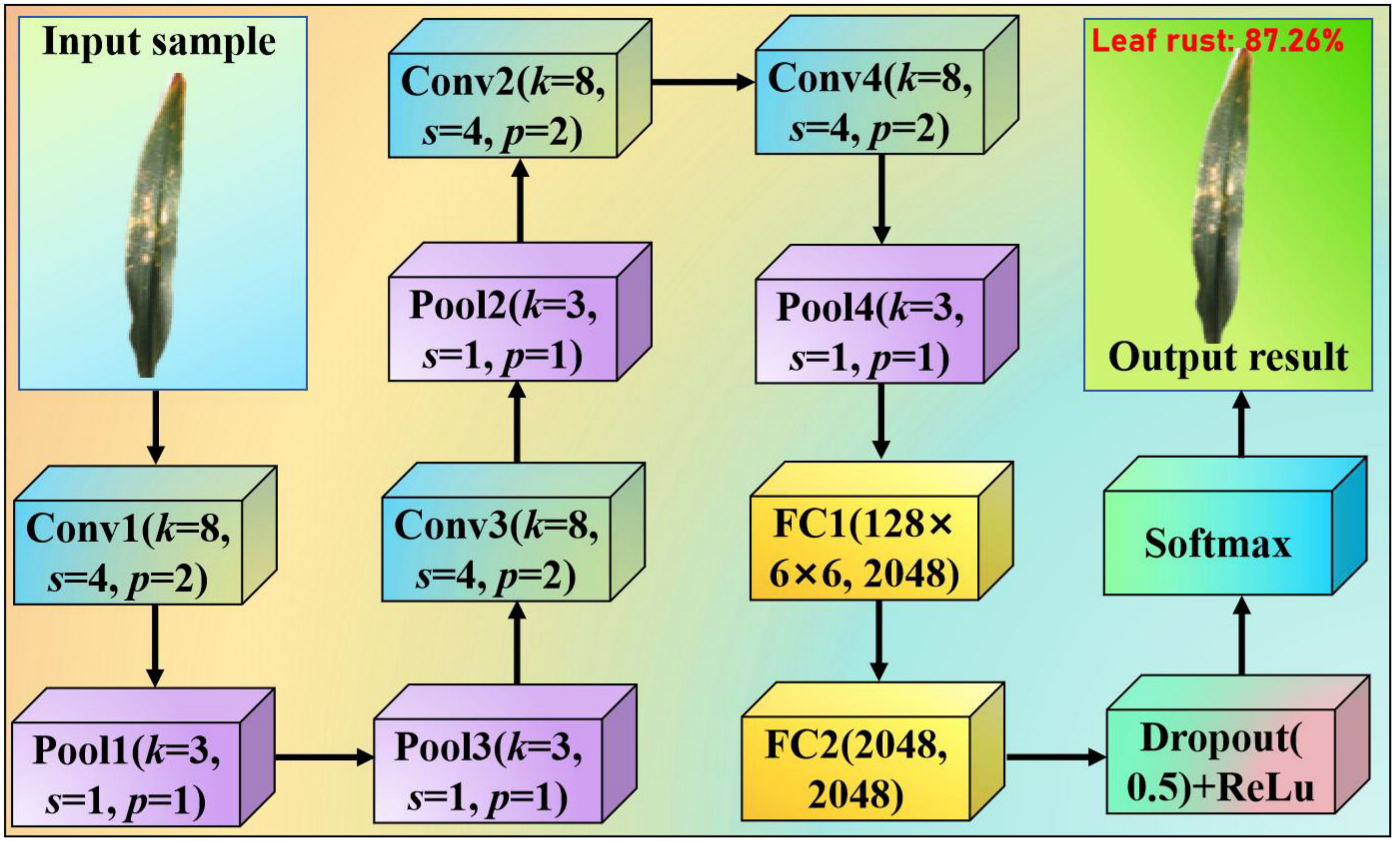
Improved AlexNet framework.

In the range of 3×3 to 8×8, the accuracy increases gradually with the increase of the receptive field, while in the range of 11×11, the accuracy decreases and the computational cost is higher. 8×8 achieves the best balance between accuracy and computational efficiency, so it is selected as the first convolution kernel size, which is combined with the subsequent 3×3 convolution to further refine the feature extraction. To address parameter redundancy in the fully connected layers of the original AlexNet, we reduced the dimensionality of these layers from 4096 to 2048. The optimized model comprises an input layer, four convolutional layers, four pooling layers, three fully connected layers, and an output layer. Herein, K, S, and P denote kernel size, stride, and padding, respectively. Detailed network architecture and parameter settings for the modified model are presented in **Figure 1**. The improved AlexNet can reduce the computational overhead and maintain good basic feature extraction ability. However, due to the reduction of the depth of the network, its perception of the small lesion and complex texture of wheat leaves is still insufficient.

### 3.2 Convolutional block attention module

Although the optimized AlexNet reduces baseline computational overhead and improves recognition accuracy to a certain extent by cutting convolutional layers, it compromises feature extraction capability due to reduced network depth. Fine lesion textures, color gradients, and other key features of diseased leaves are insufficiently captured, which limits the initial recognition accuracy. Additionally, this model still fails to completely address the feature redundancy issue that is inherent to traditional lightweight networks, leaving this critical drawback unresolved in practical applications.

**Figure 2:**
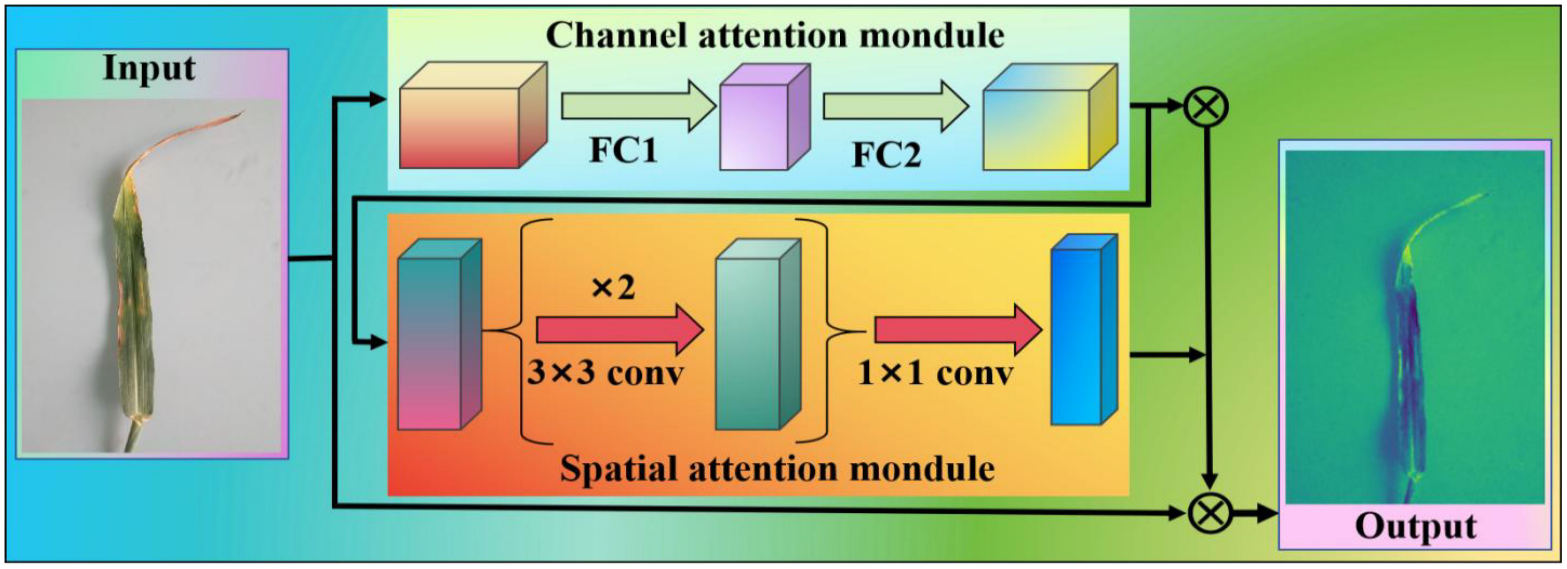
Proposed CBAM framework.

To remedy these defects, we introduce multiple CBAM attention mechanism modules between the convolution layers to improve the feature capture ability of the model. The channel attention module can accurately screen the core feature channels that are valuable for disease identification, eliminate redundant channel information, strengthen effective feature transmission without increasing the network depth, and further optimize computational efficiency. In addition, the spatial attention module can focus on the lesion area, weaken the background noise such as soil and weeds, and avoid the model learning irrelevant information due to inaccurate feature extraction, which not only improves the utilization rate of disease features but also indirectly reduces the risk of overfitting. The synergy of the two makes up for the short board of feature extraction caused by the reduction of convolution layers on the basis of retaining the advantages of lightweight architecture and finally balances the recognition accuracy and computational efficiency, which is more suitable for the actual needs of real-time detection of agricultural field diseases. The detailed structure of the CBAM module is shown in **Figure 2**.

Given an intermediate feature map $\text{F}\in R^{C\times H\times W}$ as input, CBAM derives a 1D channel attention feature map $M_{C}\in R^{C\times 1\times 1}$and a 2D spatial attention map $M_{S}\in R^{1\times H\times W}$mathematically expressed in Equation 1 and Equation 2:

(1) $$F^{\prime }=M_{C}(F)\in F$$

(2) $$F^{\prime \prime }=M_{S}(F^{\prime })\in F^{\prime }$$

where Ä is the element-by-element multiplication.

After the attention of the CBAM is placed in the convolution layer, in the process of feature extraction and learning, it is assumed that *F* is the feature input graph, *H* is the height of the input graph, *W* is the width, and *C* is the number of channels. CBAM includes the following steps:

First, the convolutional layer outputs various parameters through feature extraction to obtain the feature input graph: $F\in R^{C\times H\times W}$. In order to extract the channel features, the feature map is first pooled, and the average and maximum values of each channel are calculated.

The mathematical expression for average pooling can be described by Equation 3:

(3) $$F_{avg}=\frac{1}{H\times W}\sum_{h=1}^{H}\sum_{w=1}^{W}F[:,h,w]\in R^{C}$$

The mathematical expression for maximum pooling is defined in Equation 4:

(4) $$F_{\max}=\max(F)\in R^{C}$$

To increase the nonlinear expressiveness of the model, we introduce fully connected layers and use activation functions on average and maximum pooling results. The channel attention mapping can be computed using Equation 5:

(5) $$M_{C}=\sigma (FC_{1}(F_{\text{avg}})+FC_{2}(F_{\max}))$$

where σ is the activation function, ${\text{FC}}_{1}$ and ${\text{FC}}_{2}$ refers to the output of the fully connected layer.

Finally, the input feature map F is adjusted according to the calculated channel attention map ${\text{M}}_{\text{C}}$. It can be expressed by Equation 6:

(6) $$F_{\text{out}}=M_{C}⊙F$$

where ⊙ represents element-wise multiplication.

After processing by the channel attention module, an adjusted feature map ${\text{F}}_{\text{Out}}$ is obtained. Next, a pooling operation is needed to generate the spatial attention map. The process can be expressed as Equation 7:

(7) $$F_{avg}=\frac{1}{C}\sum_{C=1}^{C}F_{out}[C,:,:]\in R^{H\times W}$$

where ${\text{F}}_{\text{avg}}$ is averaging the channel features at each spatial location to generate an *H×W* feature map. It can be calculated as Equation 8:

(8) $$F_{\text{out}}=\underset{C}{\max}(F_{out})\in R^{H\times W}$$

Next, the results of average pooling and maximum pooling are concatenated together to generate a spatial attention map $M_{s}$. It can be described by Equation 9:

(9) $$M_{S}=\sigma (C\text{oncat}(F_{avg},F_{\max}))\in R^{1\times H\times W}$$

Finally, according to the calculated spatial attention map ${\text{M}}_{\text{S}}$, the feature map $F_{out}$ after the channel attention is adjusted, and its mathematical expression can be formulated as Equation 10:

(10) $$F_{\text{out}}=M_{S}⊙F_{out}$$

### 3.3 Squeeze-and-excitation network

**Figure 3:**
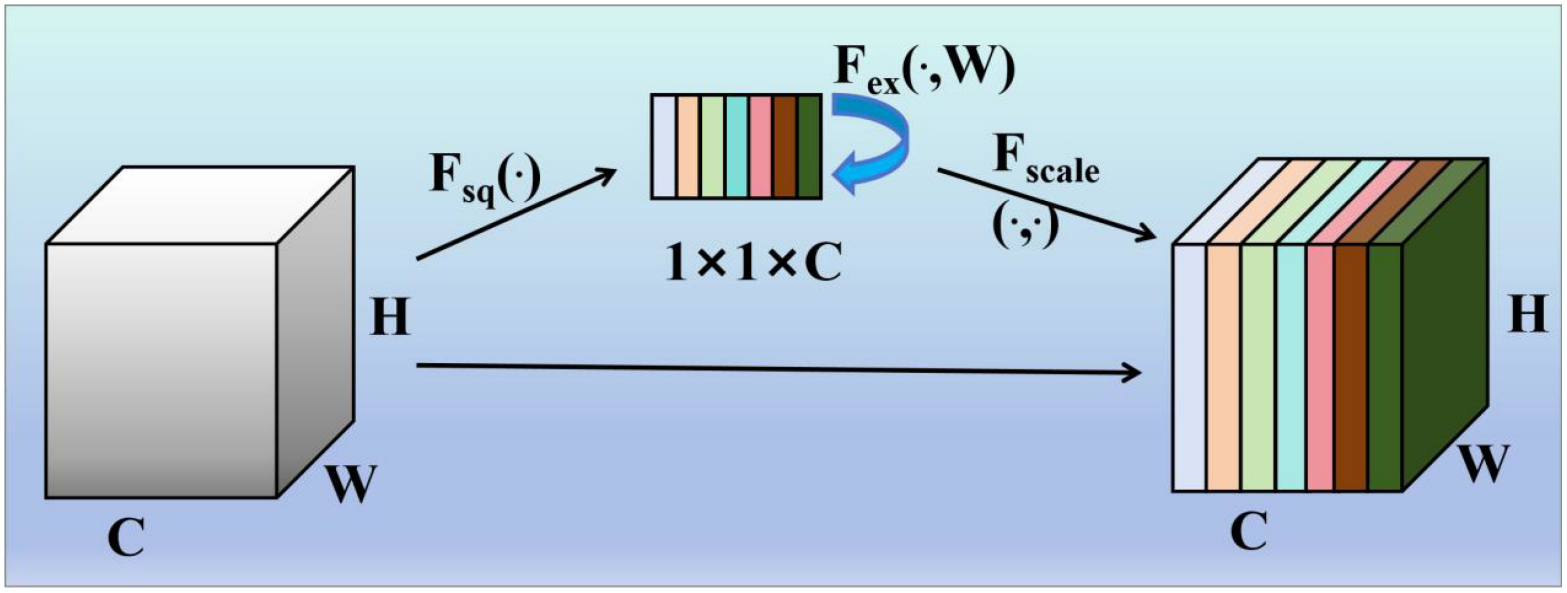
Proposed SENet framework.

The CBAM self-attention module markedly enhances the local feature extraction capability for diseased leaves and boosts single-environment recognition accuracy, yet it has two key limitations. First, varying field conditions, including light, moisture, and background interference, cause significant variations in disease features, and CBAM struggles to distinguish environmental interference features, leading to poor cross-environment generalization. Second, CBAM relies on full-scale feature association calculations, which induce invalid operations on redundant features such as healthy leaf texture and soil background, causing resource wastage and impairing real-time performance. Thus, we integrated SENet into convolutional layers, which leverages the squeeze-and-excitation mechanism to focus on channel weights with low computational overhead. The SENet design process is presented in **Figure 3**.

Image features are extracted through continuous convolution pooling, and a feature map with height H, width W, and channel number C is obtained and input into the SENet model framework.

The proposed SENet generates channel eigenvector z by global averaging pooling operation. It can be defined as Equation 11:

(11) $$\text{z}=Squeeze(X)=\frac{1}{H\times w}\sum_{i=1}^{H}\sum_{j=1}^{W}X(i,j)$$

The SENet processes channel features through fully connected layers to generate specific weights S for each channel. It can be formulated as Equation 12:

(12) $$\text{S}=F_{ex}(z,W)=\sigma (g(z,W))=\sigma (W_{2}\delta (W_{1}z))$$

where σ is the Sigmoid activation function. δ is the ReLU activation function. This equation deals primarily with eigenvector z. Finally, it uses the calculated excitation weights to recalibrate the original feature map. The process can be determined by Equation 13:

(13) $${\text{X}}_{out}=X\times \text{S}$$

The final output of 1×1×C is obtained.

### 3.4 Cross-sample statistics regularization

In the ACSE architecture, batch normalisation is introduced into the output of each convolution layer as a cross-sample statistical regularisation strategy to constrain and stabilise the feature distribution. For a mini batch containing m samples, the characteristic tensor output by batchnorm on the convolution layer calculates statistics across sample and spatial position dimensions for each channel dimension and restores the expression ability of features through learnable parameters. The specific process is as follows:

First, the mean value of the features in the mini-batch on each channel can be calculated as Equation 14:.

(14) $$\mu_{B}=\frac{1}{m\times H\times W}\sum_{i=1}^{m}\sum_{h=1}^{H}\sum_{w=1}^{W}x_{i,c,h,w}$$

where $x_{i,c,h,w}$ represents the eigenvalue of the ith sample at channel C and spatial position (h, w), and H and W are the height and width of the characteristic graph.

The variance of features on each channel can be computed as Equation 15:

(15) $$\sigma_{B}^{2}=\frac{1}{m\times H\times W}\sum_{i=1}^{m}\sum_{h=1}^{H}\sum_{w=1}^{W}{\left(x_{i,c,h,w}\mu_{B}\right)}^{2}$$

Normalizing the features based on the above statistics can be expressed as Equation 16:

(16) $${\overset{\wedge }{x}}_{i,c,h,w}=\frac{x_{i,c,h,w}\;\mu_{B}}{\sqrt{\sigma_{B}^{2}+\varepsilon }}$$

where ε is a minimum constant that prevents the denominator from being zero, usually of the order of 10^−5^.

Finally, the standardised results are scaled and offset by learnable parameters to restore the expression ability of features can be defined as Equation 17:

(17) $$y_{i,c,h,w}=\gamma_{c}\times {\overset{\wedge }{x}}_{i,c,h,w}+\beta_{c}$$

$\gamma_{c}$ and $\beta_{c}$ are the learnable scaling factors and offsets corresponding to channel C, respectively, allowing the model to adaptively adjust the centre and scale of feature distribution to avoid the damage to normalisation of feature expression ability.

Embedding batch normalisation (BatchNorm) after each convolution in our ACSE constrains layer inputs to a stable range around zero mean and unit variance, mitigating internal covariate shift and reducing gradient fluctuations, thereby lowering sensitivity to initialisation and learning rate. Concurrently, dropout randomly discards neurons during training, preventing over-reliance on single units and promoting robust distributed representations. These two regularisations are complementary: BatchNorm stabilises feature distributions, while dropout weakens neuron-level dependency, together forming a dual-level mechanism that enhances generalisation.

**Figure 4:**
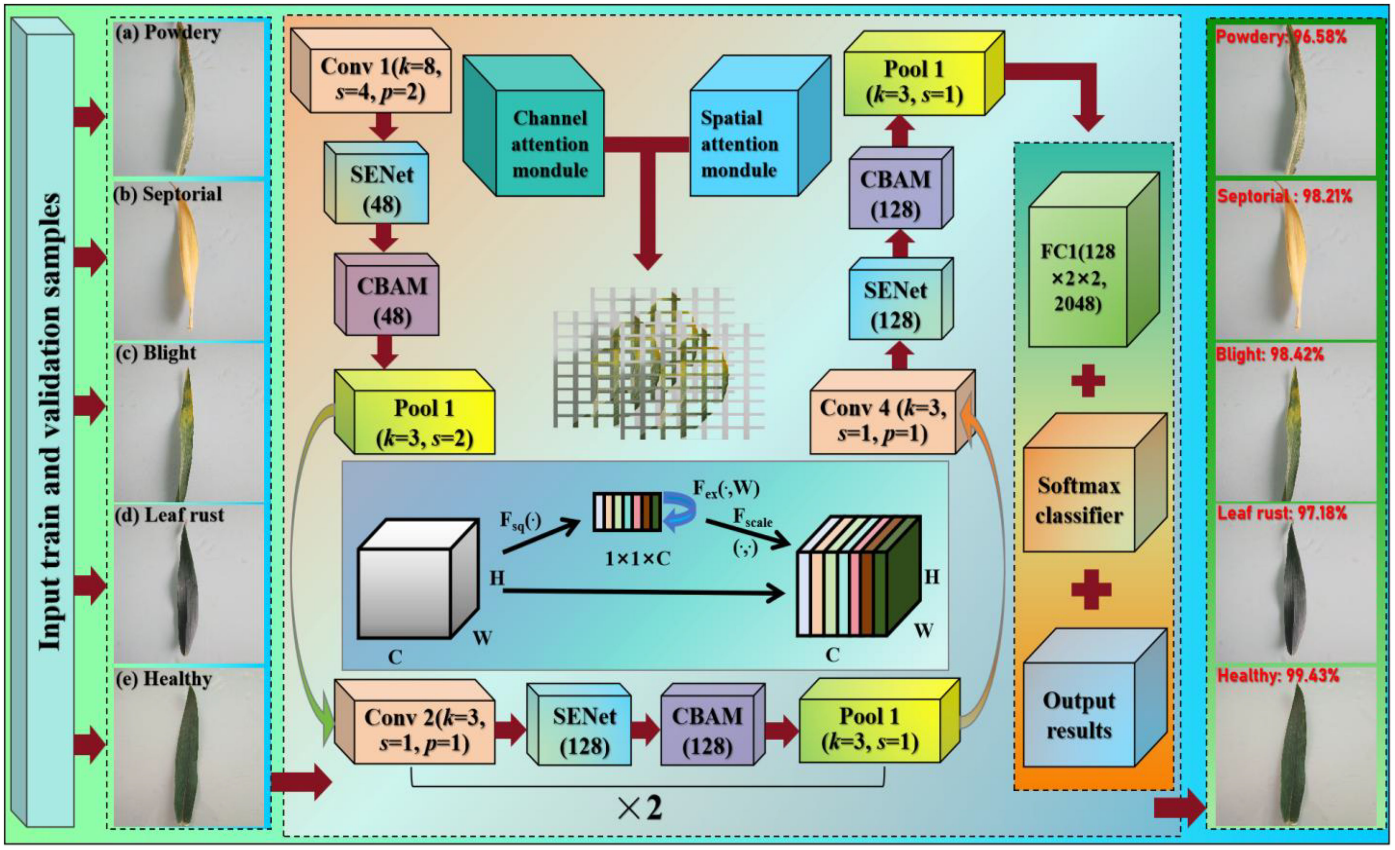
Proposed ACSE framework.

Based on the above analysis, the proposed overall ACSE framework for wheat leaf disease identification is presented in **Figure 4**.

The architecture shows the complete data flow from the original wheat leaf image to the final classification result. Wheat leaf images, following batch preprocessing, are first passed through an initial 8×8 convolutional layer to extract low-level visual features such as edges and textures. Subsequent convolutional submodules implement a hierarchical processing pipeline: feature extraction, batch normalization, channel-wise attention via SENet, non-linear activation, spatial attention via CBAM, and 3×3 max-pooling with dropout to mitigate overfitting. SENet dynamically recalibrates channel-wise feature responses, emphasizing disease-specific spectral signatures (e.g., lesion color contrast), while CBAM refines spatial focus by suppressing background interference from soil and weeds. In the overall architecture of our ACSE, within each convolution block, the senet and CBAM modules are successively embedded after the relu activation function and before the pooling layer, and the data flow follows the order of conv, batchnorm, relu, senet, CBAM, and pooling. Senet is responsible for the feature recalibration of the channel dimension of the convolution output, and CBAM then introduces spatial attention on this basis to achieve spatial focus after channel screening, forming a synergy rather than a simple parallel stack. After testing, the order of SENet in front and CBAM in back in the current architecture is focused on the space after channel screening, and its performance is better than other arrangements.

**Figure 5:**
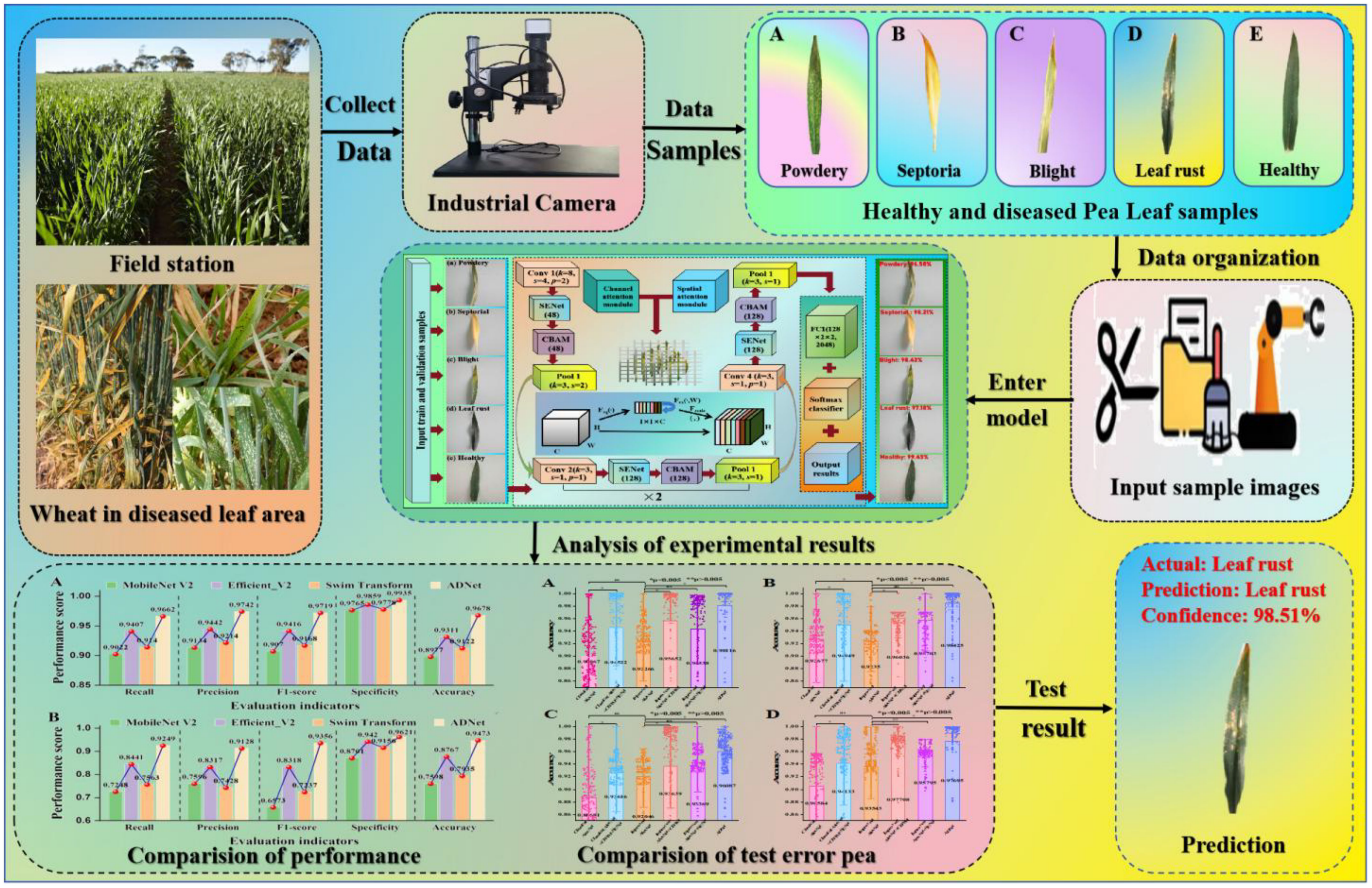
Proposed overall wheat leaf disease identification system.

The integrated architecture has been verified on a self-built dataset containing 7390 images. See Section 4.2 for the specific composition and division of the dataset. The model achieved 98.51% classification accuracy on the test set. The end-to-end workflow from field collection to model reasoning is shown in **Figure 5**.

The whole system of wheat leaf disease recognition proposed in this paper consists of three main stages: using industrial cameras to collect field images, completing model reasoning through the ACSE architecture, and finally outputting disease classification results. In the figure, the four diseases and health categories are easily distinguished by visual markers, and the corresponding comparative experimental results are attached.

## 4 Experimental results and analysis

### 4.1 Experimental setup

Experimental software and hardware are described in **Table 2**.

**Table 2:** Experimental software and hardware.

Operating system	Windows 11 (64-bit)
Processor	AMD Ryzen 9 7945HX
Graphics card	NVIDIA GeForce RTX 4060 Laptop GPU
Memory	16 GB
Deep learning framework	PyTorch
Editor	PyCharm 2024.1
Programming language	Python 3.9

### 4.2 Data description

**Figure 6:**
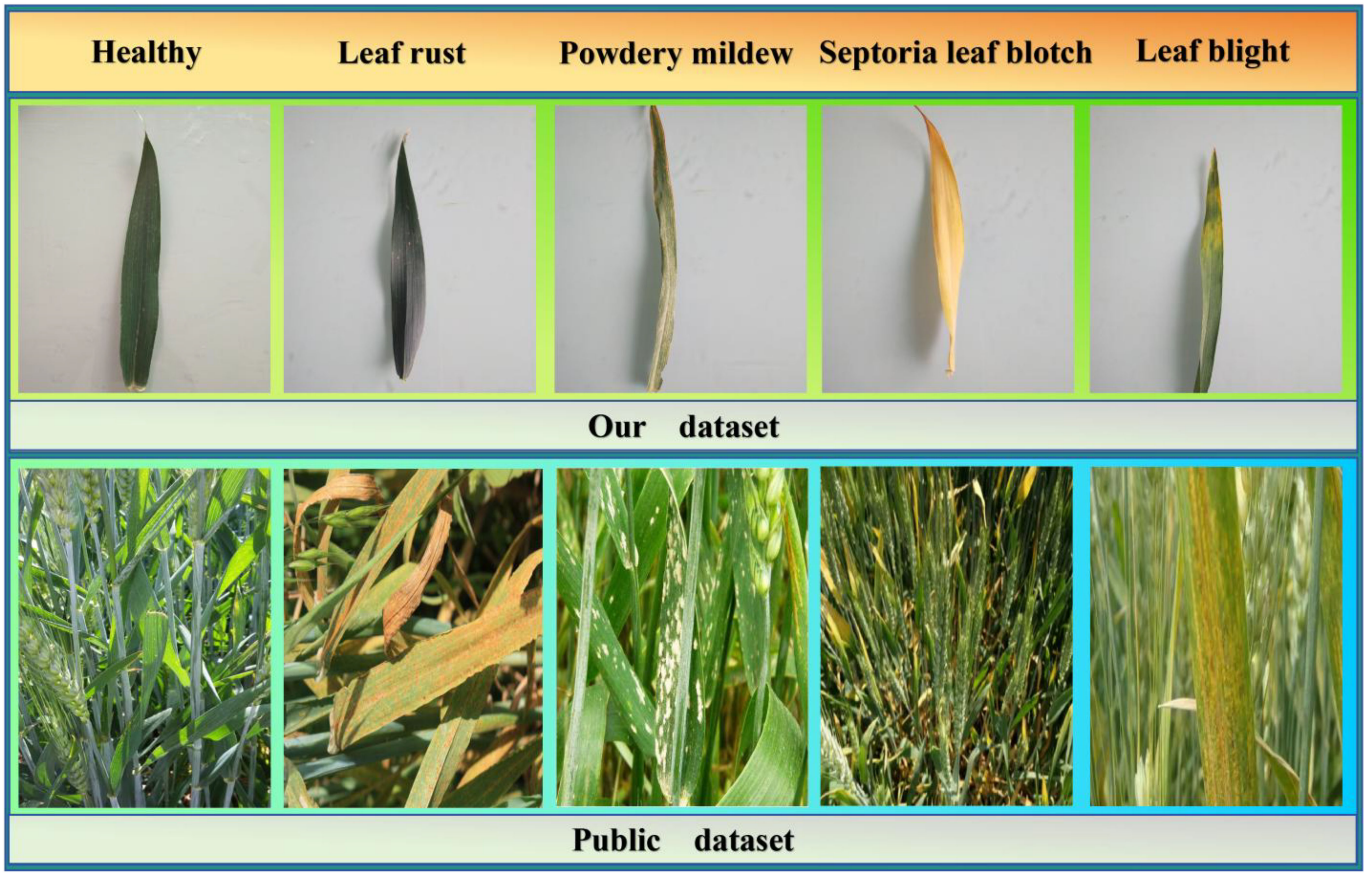
Wheat leaf disease samples.

Our dataset comprises 7, 390 samples, captured by industrial cameras in a wheat plantation in Henan Province, China, and partially generated via the Pix2Pix generative adversarial network. Among them, the total number of samples generated by pix2pix is about 2000, accounting for about 27% of the total number of self-built data sets. The generated samples cover all four disease and health categories, and the generated quantity of each category is consistent with the proportion of the real sample to ensure that the category distribution deviation is not introduced. All generated images are manually screened one by one to eliminate unqualified samples with obvious artefacts or texture distortion. The data set covers four wheat leaf diseases (powdery mildew, leaf blight, Septoria leaf blotch, and leaf rust) and healthy leaves. In addition, we collected 2000 public data samples of the same category as our data to verify the generalization ability of our model. The public dataset was obtained from LWDCD2020 of Informatics in Medicine Unlocked. Its download link is https://aistudio.baidu.com/dataset/detail/231774/intro. In terms of preprocessing, the publicly available dataset images adopt the same standardisation process as self-built data, including resizing to 224 by 224 pixels and normalising pixel values. The label mapping rules follow the category definitions provided by the original dataset and correspond to each of the four disease and health categories in our experiment. Our and public datasets are presented in **Figure 6**.

Healthy wheat leaves are bright green or dark green. The leaves are stretched and straight, without curl, shrinkage, or scorch. The texture is soft and tough. The main manifestations of wheat leaf powdery mildew are small yellowish spots on the leaves, covered with a layer of white and loose powder, which can lead to the death of the whole plant in serious cases. Leaf blight is characterized by rapid withering of the whole leaf, which is yellowish brown to grayish brown. The leaves are curly and easy to break. In severe cases, the whole plant’s leaves wither at the same time. However, Septoria leaf blotch is mainly caused by yellowish-brown, near-circular or oval spots on the leaves, and multiple spots can be connected into pieces, leading to the drying of the leaves from the tip to the base. Wheat leaf rust mainly developed from raised spots into round or oval summer spore mounds, which were reddish brown or yellowish brown, with a diameter of 0.5~1 mm. At the same time, the leaf tissue was damaged by spore mounds and gradually faded green and yellow. In this study, multi-source cross-validation was used for label validation. First, the researchers of our research group collected leaf samples in the field and preliminarily classified them according to the visible symptoms and then compared the sample image with the standard wheat disease map one by one. To ensure the accuracy of labelling, we consulted professionals with many years’ experience in agricultural means of retail and farmers who planted wheat on a large scale and reviewed the labelling results with the help of their field practice experience. For individual samples with vague symptoms or in the early stages of the disease, further consult the plant pathology data to compare the morphological characteristics as an auxiliary basis for label determination. The sample category label of the public dataset is subject to the annotation information provided by the original source and has not been modified.

Given the moderate dataset size, a 3:1:1 ratio allocated 7390 wheat leaf images into training (4434), validation (1478), and test (1478) sets. In order to ensure the independence of the evaluation, the data is divided into batches rather than randomly at the level of a single image. All images collected in the same batch are only assigned to a subset of the training set, validation set, or test set, and the cross-collection of highly similar images from the same plant or adjacent plots is blocked from the source. For the extended samples generated by pix2pix, each original real image and its corresponding generated variant always belong to the same subset, and there will be no separation between the training set and the test set. The publicly obtained samples and self-built data sets are independent of each other, and there is no intersection. The test set is all composed of real field-collected images, and the training set and validation set are mixed with self-collected images, generated samples, and public data. This partitioning ensures sufficient training samples for feature learning while maintaining statistical reliability. The validation set enables hyperparameter tuning and overfitting prevention during training, while the test set provides unbiased generalization assessment. Random division with fixed seeds guarantees reproducibility and mitigates partitioning bias. Category distributions across sets remain balanced as detailed in **Table 3**, supporting rigorous model evaluation and result interpretability.

**Table 3:** Division of experimental data.

Category	Training set	Validation set	Test set	Total size
Powdery mildew	912	304	304	1520
Septoria leaf blotch	807	269	269	1345
Leaf blight	906	302	302	1510
Leaf rust	783	261	261	1305
Healthy	1026	342	342	1710
Total size	4434	1478	1478	7390

### 4.3 Train and test results

In order to determine the optimal hyperparameter configuration of the model, we designed a set of systematic parameter optimization experiments. For the ACSE model, we evaluated its test loss and test accuracy under a variety of superparameter combinations. The main parameters adjusted include batch size and initial learning rate. The weight initialization strategy is also included in the comparison. The number of training rounds is fixed at 50. During the experiment, the training dynamics and verification performance corresponding to each set of parameters were recorded in detail, and the results clearly reflected the significant impact of super-parameter selection on the model effect. Detailed experimental data are summarized in **Table 4**.

**Table 4:** Experimental parameter configuration.

Method	Batch size	Initial learning rate	Weight	Epoch	Test loss	Test accuracy
ACSE	16	0.01	0.001	50	0.078	0.9783
	16	0.001	0.01	50	0.069	0.9764
	32	0.01	0.01	50	0.077	0.9756
	32	0.001	0.01	50	0.028	0.9834
	32	0.001	0.001	50	0.021	0.9851
	32	0.001	0.004	50	0.035	0.9805
	32	0.0003	0.004	50	0.069	0.9796
	64	0.01	0.004	50	0.047	0.9772
	64	0.001	0.004	50	0.094	0.9663

The test performance of our proposed model is affected by the batch size, initial learning rate, and weight. There is no single optimal parameter, which needs to be optimized according to the combination of the three. It can also be seen from **Table 4** that when the learning rate is 0.0003 and the weight attenuation is 0.004, the test accuracy rate drops to 0.9796, which is significantly lower than most configurations. The relatively high value of weight attenuation relative to the learning rate in this group of parameters may lead to excessive regularization intensity and inhibit effective feature learning, reflecting that the interaction between the two cannot be ignored. This phenomenon also shows that the norm constraint imposed by weight attenuation in the parameter space and the distribution constraint imposed by batchnorm in the feature space do not work in isolation, and only when they work together can they form a more robust regularization system. On the whole, the optimal configuration obtained by parameter optimization improves the recognition accuracy of the model by 1.88% and reduces the loss function by 7.3% compared with the lowest recognition accuracy of other parameter groups. It shows good overall performance, and the accuracy of all parameter identifications is more than 96%. The accuracy of the constructed deep learning model meets the requirements of wheat leaf disease identification.

On the basis of completing the super parameter search, all experiments follow the unified fixed training settings, as shown in **Table 5**. Among them, the optimizer chooses AdamW because it combines adaptive learning rate with decoupling weight attenuation and can give consideration to convergence speed and generalization performance under limited training rounds and medium-sized data. All models are trained with a fixed 50 epochs and do not use the early stop strategy so as to ensure that each model completes the comparison under the same training budget and avoid the introduction of evaluation bias in the early stop system due to the different convergence rhythms of different models. The random seed is uniformly fixed to 42, which completely covers all random operations in data division, weight initialization, and data enhancement. The data enhancement in the training phase includes random horizontal flipping, color jitter (brightness factor 0.3, contrast factor 0.3, saturation factor 0.3), random rotation (maximum angle 20 degrees), and random affine transformation (shear factor 0.2) so as to simulate the possible illumination change and angle shift in field shooting. The above settings apply to both our ACSE and all comparison models.

**Table 5:** Fixed training protocol.

Setup item	Value or description
Optimizer	AdamW
Weight attenuation coefficient	Corresponding to **Table 3**, take 0.001, 0.004, or 0.01.
Loss function	Cross-entropy loss
Learning rate scheduler	Not used, constant learning rate
Number of training rounds	Fix 50 epochs.
Random seed	Fixed to 42
Model checkpoint selection rules	Select the corresponding parameters of epoch with the highest accuracy in the validation set.
Data enhancement strategy	Random horizontal flip, color jitter, random rotation, random affine transformation, standardization
Scope of application	ACSE and all comparison models

**Figure 7:**
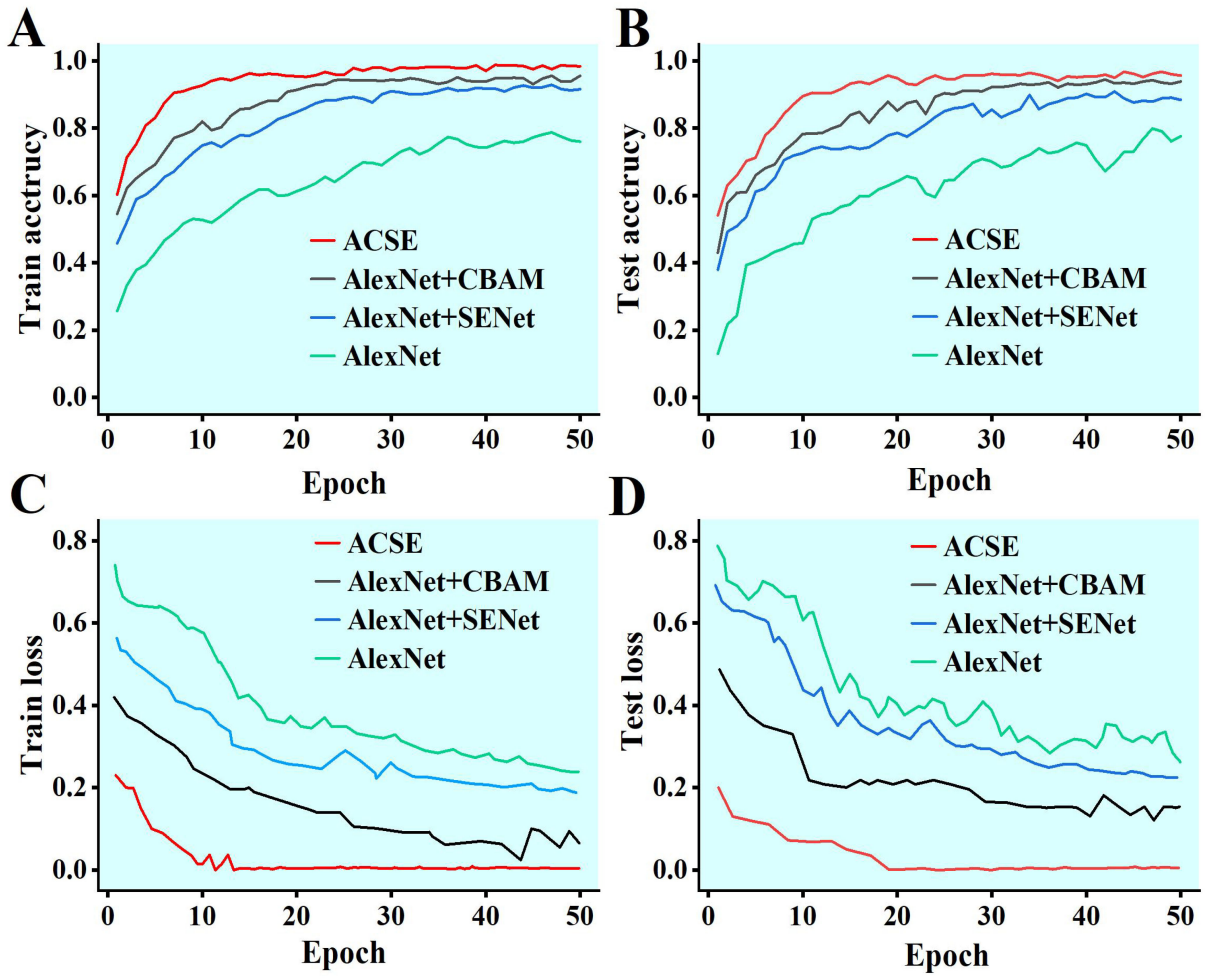
Accuracy and loss value change curves. **(A)** is the training accuracy. **(B)** is the test accuracy. **(C)** is the training loss. **(D)** is the test loss.

To compare the training effectiveness of different models, we selected the classic AlexNet architecture to compare with the proposed ACSE model to evaluate the performance difference between the two in the task of wheat leaf disease recognition. In order to comprehensively compare the learning dynamics and convergence behavior of the model, we take the training accuracy and training loss value as the main evaluation indexes. The performance curves of the two models during training are shown in **Figure 7**.

Detailed information has been acquired in **Figure 7**. Our ACSE model exhibited stable training accuracy, improving from 0.614 to 0.98-0.99, with loss reduced from 0.234 to 0.01-0.05. For AlexNet embedded with CBAM, accuracy rose from 0.447 to 0.91-0.93 and loss decreased from 0.45 to 0.2-0.25; with SENet, accuracy increased from 0.56 to 0.93-0.97 and loss declined from 0.41 to 0.05-0.15. In contrast, classic AlexNet accuracy only improved from 0.27 to 0.8-0.85, with loss reduced from 0.75 to 0.35-0.40. These results indicate that SENet enhances model performance more effectively than CBAM, and the ACSE architecture demonstrates superior feature extraction, convergence, and recognition accuracy, validating the methodology.

**Figure 8:**
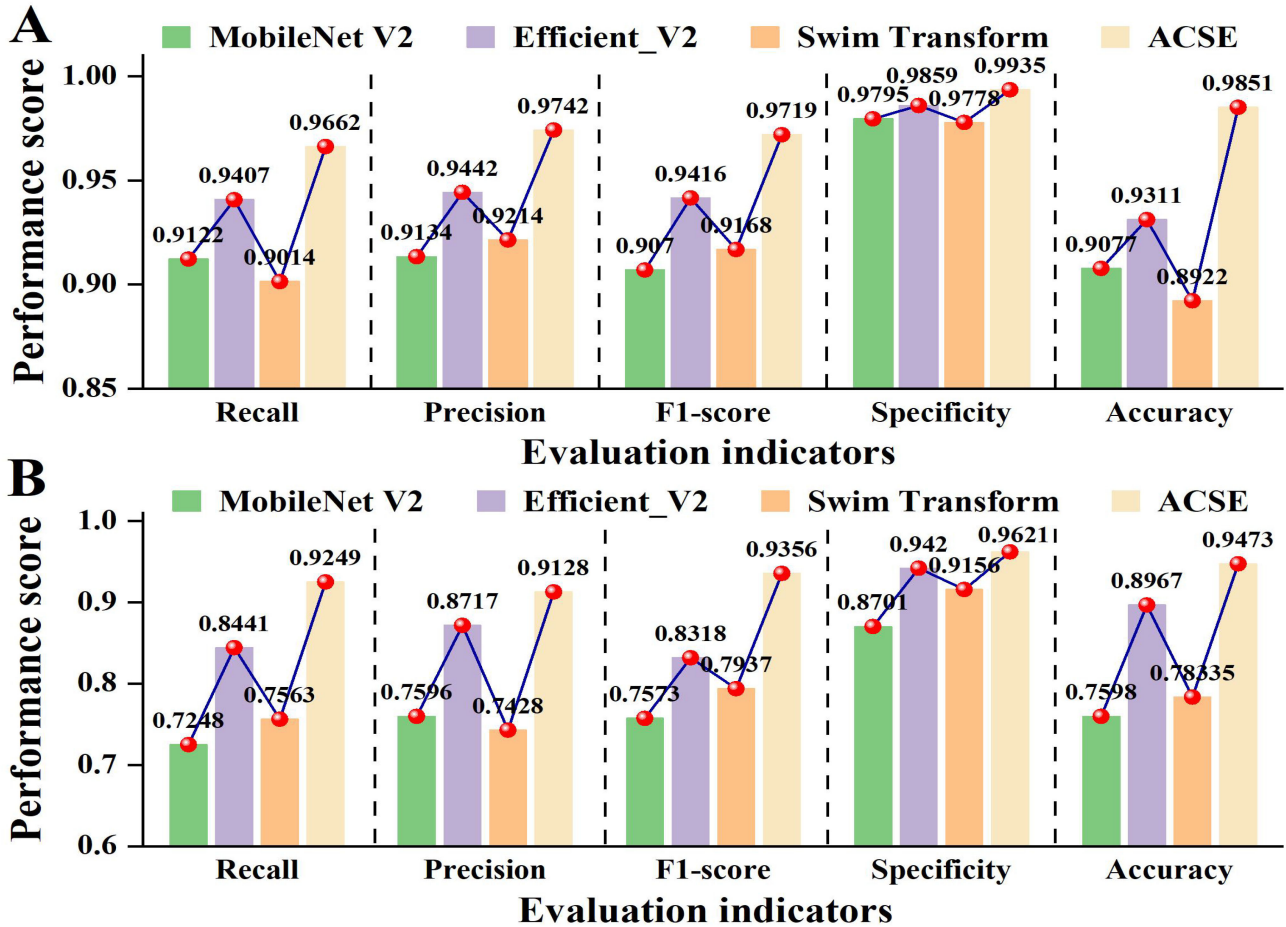
Comparisons of evaluation indicators among different models. **(A)** is on our dataset. **(B)** is on the public dataset.

To comprehensively evaluate the recognition performance of the proposed method, we selected MobileNetV2, EfficientNetV2, and Swin Transformer as the comparison method and systematically compared them with the proposed ACSE model on the five indicators. Experiments were conducted on the self-built wheat leaf dataset and the open wheat leaf dataset to verify the generalization ability of the model under different data sources. The detailed experimental results are presented in **Figure 8**.

As shown in **Figure 8**, the proposed ACSE model outperforms three advanced deep learning models across five metrics on both open-source and self-collected wheat leaf disease datasets. On the open-source dataset, ACSE exceeds MobileNetV2 by 20% in recall and EfficientNetV2 by 8.08%, while precision surpasses these models by 15.32% and 8.11% respectively. F1-score, specificity, and accuracy show 17.83%, 9.2%, and 18.75% improvements over MobileNetV2. On the self-collected dataset, ACSE maintains 2.55% higher recall than EfficientNetV2 and achieves 0.76-1.57% specificity gains, demonstrating robust cross-dataset performance. In addition, since the test set is completely composed of real field-collected images, the above results show that the generalization ability of our ACSE in real scenes is reliable, and the introduction of generated samples does not lead to overestimation of model performance.

**Figure 9:**
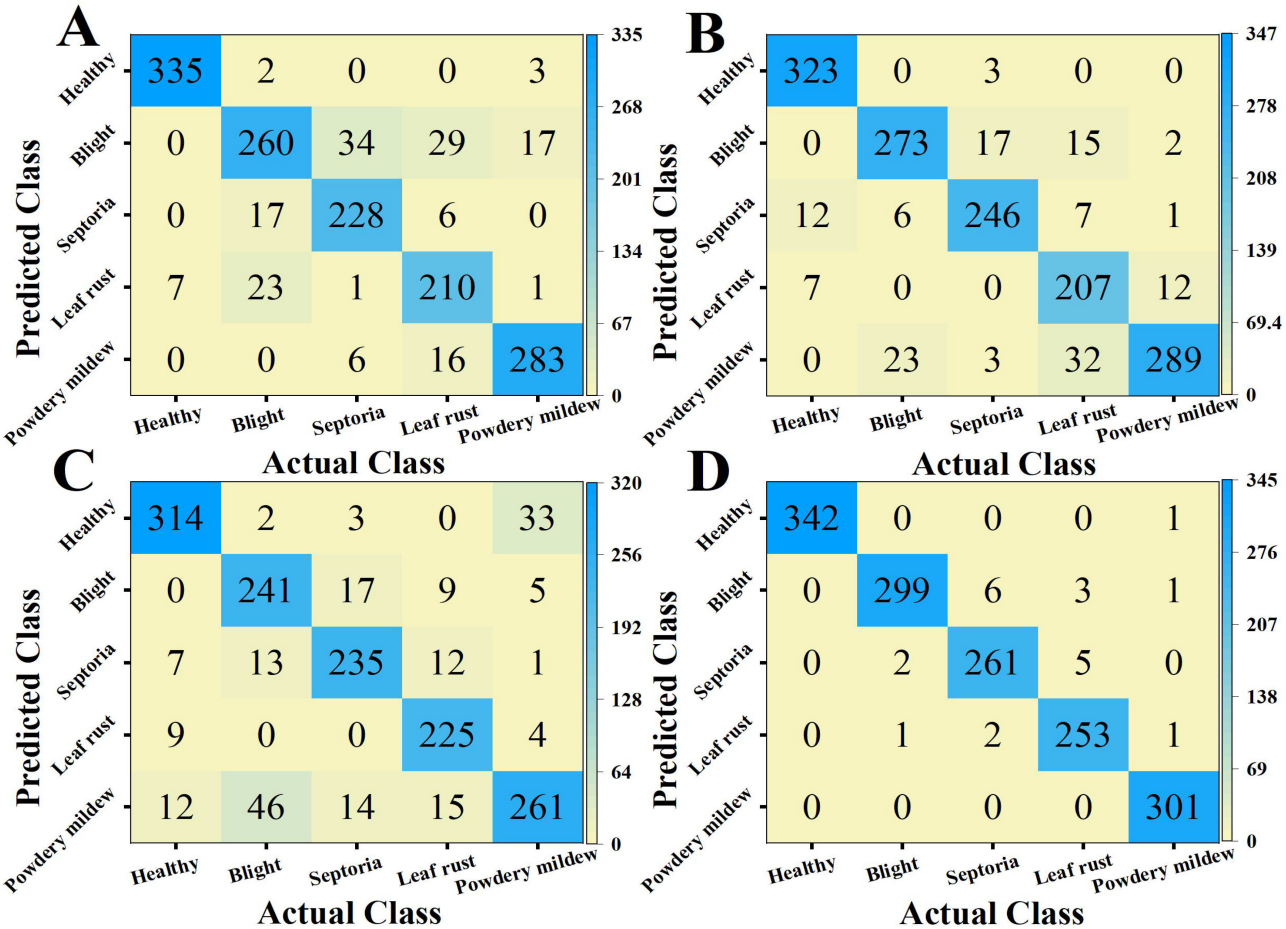
Comparisons of confusion matrices for different models. **(A–D)** are the classification and distribution of MobileNetV2, EfficientNetV2, Swin Transformer, and our ACSE models are represented, respectively.

The confusion matrix is a vital classification evaluation tool, systematically presenting the correspondence between predicted and true labels in a matrix. It reveals model strengths and error modes across categories through numerical distributions, offering a basis for optimization. To analyze model behavior, we compared MobileNetV2, EfficientNetV2, Swin Transformer, and our ACSE using the confusion matrix. The results in **Figure 9** clearly show each model’s performance characteristics in wheat leaf disease classification.

Some important observations can be made from the content of **Figure 9**. We can calculate the classification accuracy of each model: MobileNetV2 is 89.04%, EfficientNetV2 is 90.53%, and Swin Transformer is 86.33%, while the proposed ACSE model reaches 98.51%. Generally speaking, most models have poor recognition performance for Leaf blight and powdery mildew. The experimental results show that the accuracy of our ACSE is 11.82% higher than that of Swin Transformer, which is the lowest, and 7.62% higher than that of EfficientNet V2, which is the best. It shows that the model performs best in the task of wheat leaf disease classification and has high classification accuracy. It should be noted that the zero value of the non-diagonal position in the confusion matrix indicates that all samples of the corresponding category have been correctly classified and no misclassification has occurred. The smaller the number, the more it can further reflect the high discrimination reliability of the model on different blade categories.

**Figure 10:**
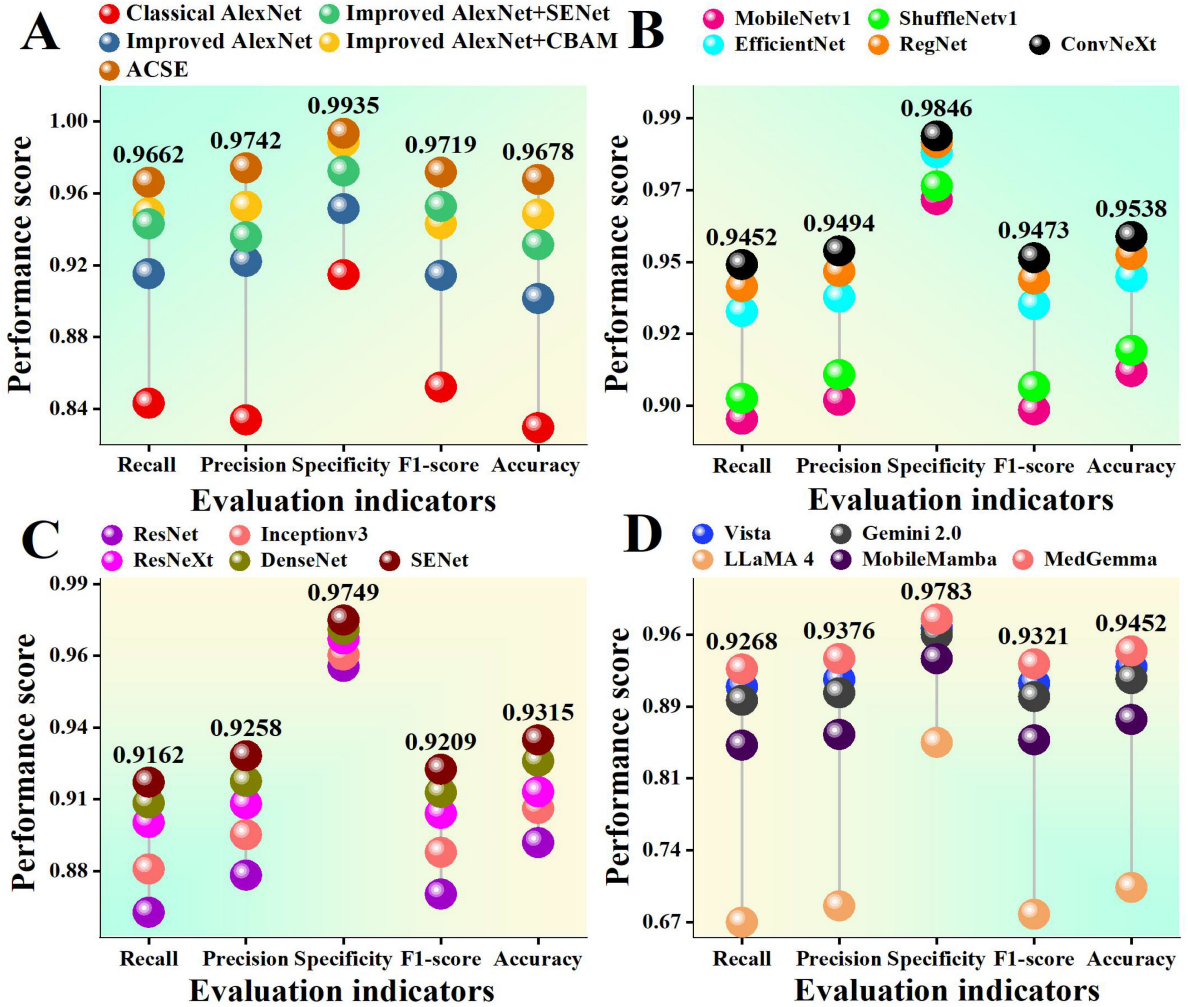
Comparisons of different models. **(A)** is the ablation experimental results of our proposed model. **(B–D)** show the performance of different models on the wheat leaf disease data set we collected.

To further clarify the contribution of each module to the performance of the model, we used a verification framework combining an ablation experiment and a comparative experiment to systematically evaluate the impact of different structures on the overall performance of the model. All experiments were carried out under the unified hardware environment and data enhancement strategy to ensure the repeatability and comparability of the results. The results are sketched in **Figure 10**.

As can be seen from **Figure 10A**, demonstrates that integrating CBAM or SENet modules into AlexNet enhanced classification performance. At the same time, **Figure 10a** also shows the test results of AlexNet optimised in Section 3.1 but without an embedded attention module. Although the baseline model has been better than the classic AlexNet in structure, the recognition ability of disease characteristics under complex field backgrounds is significantly weaker than the variants after the introduction of the attention mechanism. CBAM improved anti-interference and feature representation via spatial and channel dimensions, increasing accuracy and specificity. SENet enhanced generalization through channel recalibration, particularly boosting specificity and F1-score. In addition, we also conducted a comparative test on the arrangement order of Senet and CBAM in Ablation Experiment A. The results showed that the current configuration of Senet before CBAM was superior to the reverse arrangement in all indicators, which verified the rationality of the sequence design of channel screening first and then spatial focus. The proposed ACSE combined both mechanisms, achieving optimal recall, accuracy, specificity, and F1-score, confirming its efficacy for wheat leaf disease recognition. Marked data indicated the highest accuracy. **Figures 10B–D** revealed ConvNeXt outperformed MobileNetV1 and ShuffleNetV1 with 98.46% recall, while our ACSE ranked first across all evaluation metrics.

**Figure 11:**
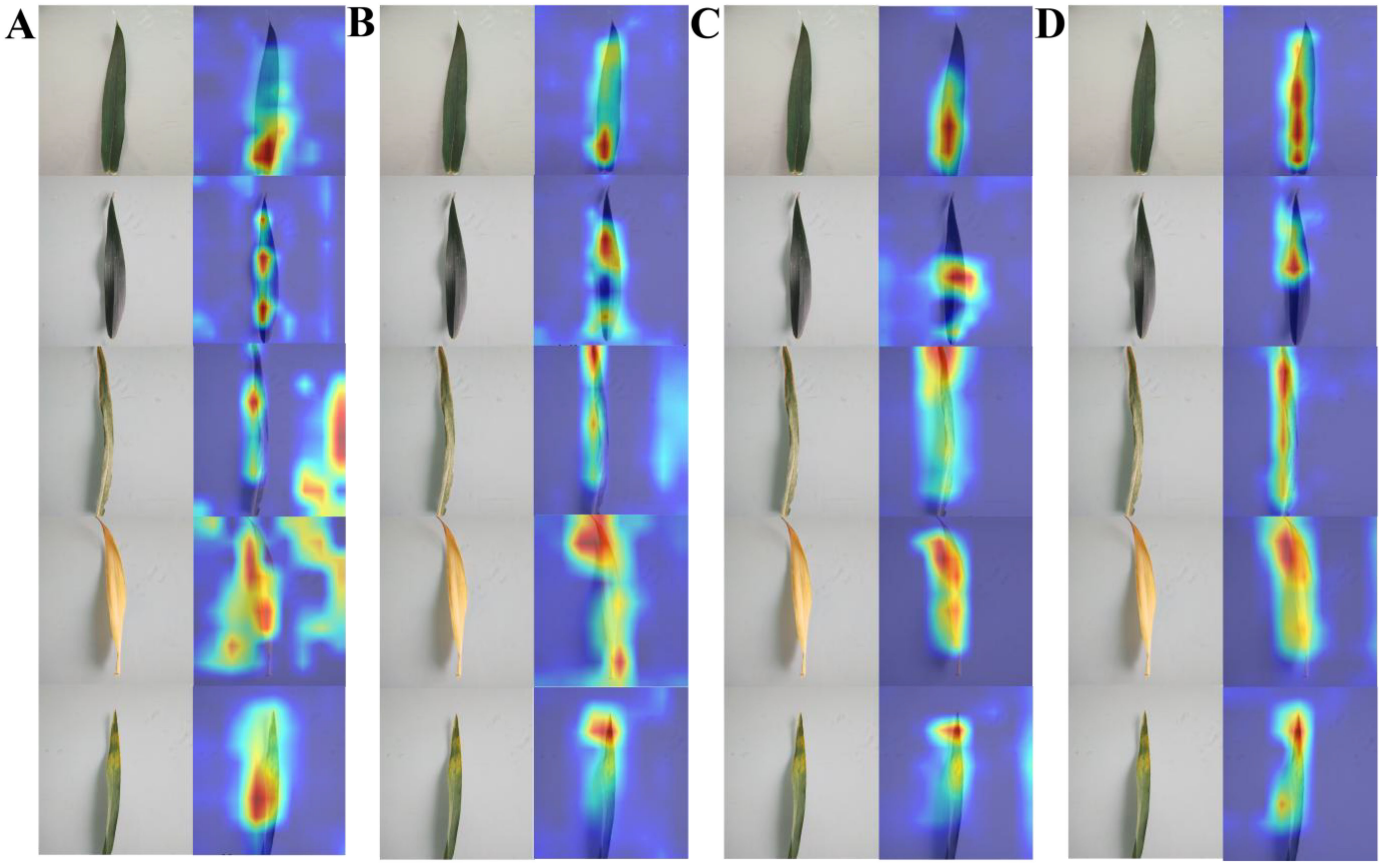
Grad-CAM attention heat maps of wheat leaf diseases under different model configurations. **(A)** is after the first convolution. **(B)** is after the first pooling. **(C)** is after the second convolution. **(D)** is after the second pooling.

In order to verify the guidance effect of CBAM and Senet on the region of interest of the model, we used Grad-CAM to visually compare the ACSE and ablation variants. Grad-CAM extracts the feature map of the convolution output of the last layer and uses the class gradient for weighted summation to generate the thermal map corresponding to the input image space. The highlighted area is the image position that the model pays most attention to when making classification decisions. In the experiment, representative samples of powdery mildew, leaf blight, conifer leaf blight, and leaf rust were selected, and attention heat maps were generated under four configurations: classic AlexNet, CBAM-only variant, SENet-only variant, and ACSE complete model. The results are shown in **Figure 11**. (Grad-CAM attention heat map of wheat leaf diseases under different model configurations. From left to right are Classic AlexNet, the CBAM-only variant, the SENet-only variant, and ACSE. From top to bottom, they represent five wheat image categories: health, leaf rust, powder mildews, Septoria leaf blotch, and leaf blight.) It can be observed from **Figure 11** that the thermal distribution of classic AlexNet is relatively diffuse, and some highly activated areas deviate from the actual location of the lesion and are scattered on the background or healthy leaf tissue, indicating that its decision-making is easily disturbed by irrelevant background information. After the introduction of CBAM alone, the thermal response region began to converge to the edge of the lesion, and the scope of attention of the model was significantly narrowed. After the introduction of senet alone, the response of the high activation area in the core of the lesion was further enhanced, and the thermal coverage was more stable. The thermodynamic map of the complete ACSE model shows the most accurate spatial positioning ability. The highly activated area closely covers the lesion and its marginal transition zone. which is highly consistent with the distribution of the actual visible symptoms. This gradual improvement trend from dispersion to focus confirms that the collaborative integration of Senet and CBAM can effectively guide the model to focus on the lesion areas directly related to the disease, rather than relying on unrelated background clues.

**Figure 12:**
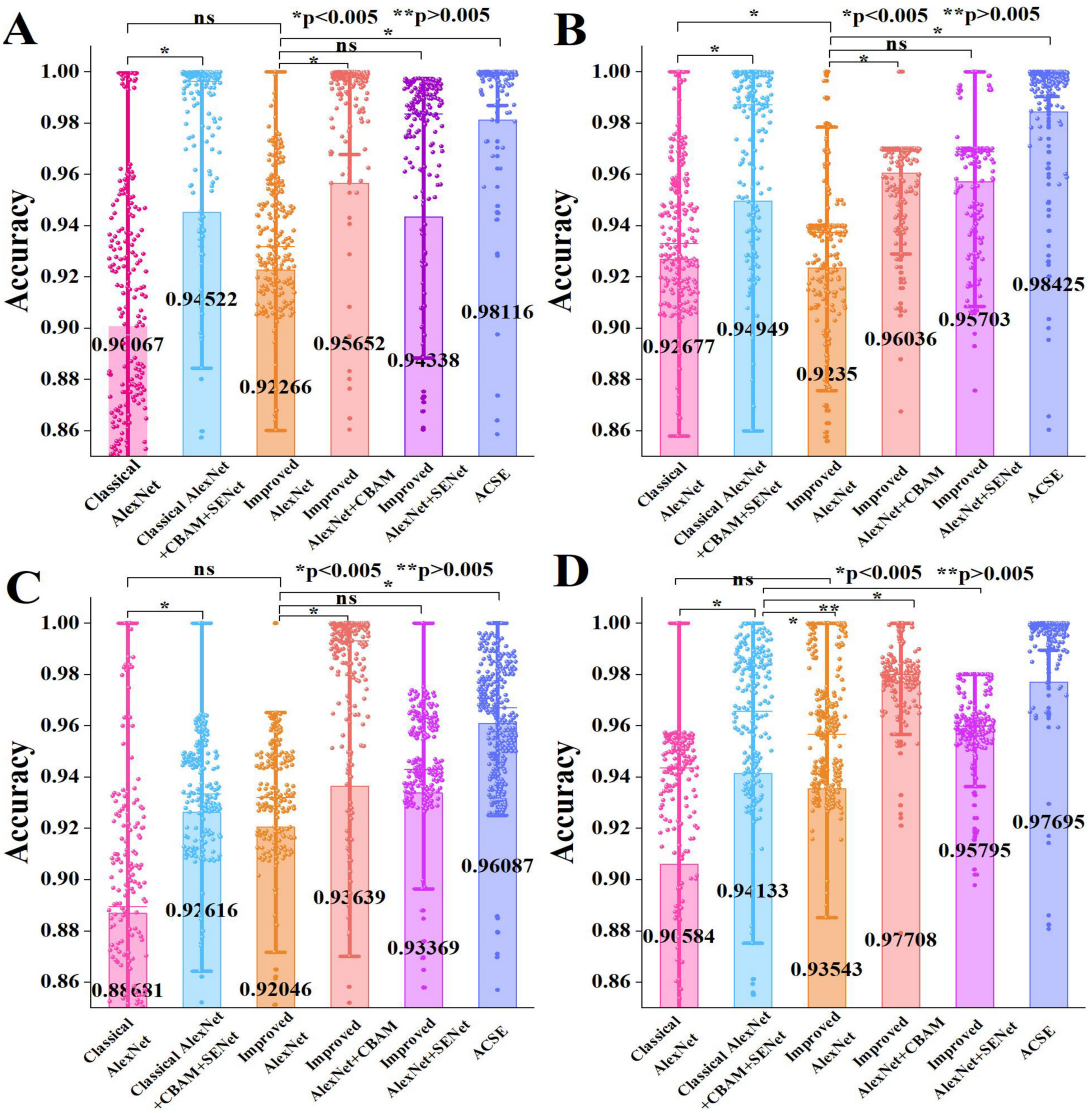
Columnar scatter diagram of four wheat leaf disease identifications. Average accuracy exceeded 90.067% for Leaf blight **(A)**, 92.677% for Leaf rust **(B)**, 88.681% for Septoria leaf blotch **(C)**, and 90.584% for powdery mildew **(D)**.

The columnar scatter diagram was used as a composite visualization tool to systematically compare the performance of the proposed ACSE model and several benchmark models in the task of identifying four types of wheat leaf diseases (e.g., powdery mildew, leaf blight, Septoria leaf blotch, and leaf rust). The experimental results are depicted in **Figure 12**. As can be seen from **Figure 12**, demonstrates that the ACSE model achieved the highest average accuracy in Leaf blight identification, surpassing both AlexNet-CBAM and AlexNet-CBAM-SENet variants, while classic AlexNet performed lowest. For Leaf rust, our ACSE attained 0.98425 accuracy, exceeding other models by up to 6.075%. In Septoria leaf blotch recognition, our ACSE outperformed the lowest-performing classic AlexNet by 7.406%. Powdery mildew analysis revealed marginal differences between improved AlexNet-CBAM and ACSE (0.013%), though significant variance occurred with AlexNet-SENet. Collectively, our ACSE maintained superior accuracy across four diseases, confirming its robust versatility for wheat leaf disease classification.

**Figure 13:**
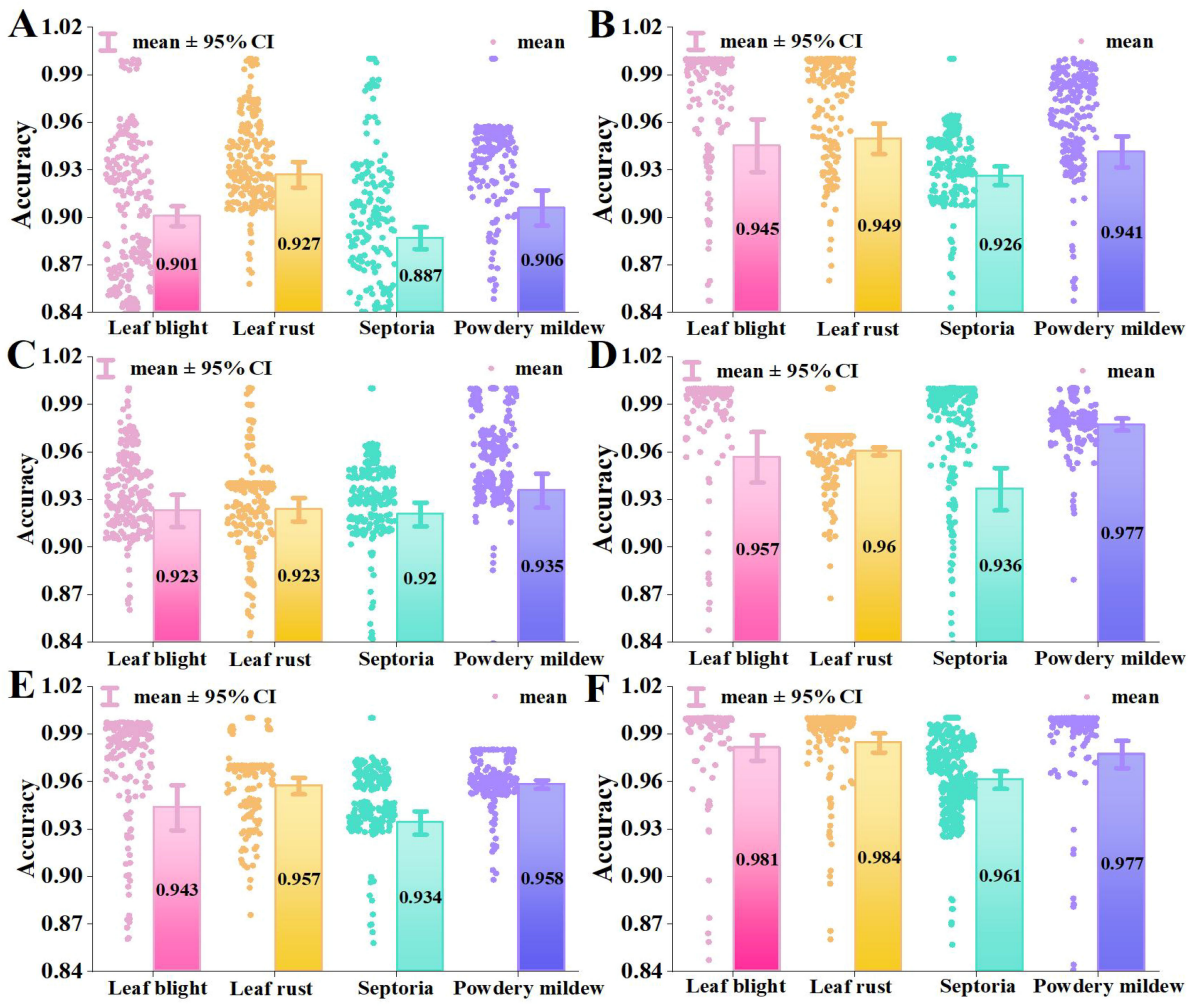
Compares the average errors of the six methods. Models a to f represent different AlexNet variants, and the average classification confidence for the four diseases ranges from 88.7% (The classic AlexNet) to more than 98.1% (Our ACSE). **(A)** is the classical AlexNet. **(B)** is the classical AlexNet + CBAM+SENet. **(C)** is the improved AlexNet. **(D)** is the improved AlexNe + CBAM. **(E)** is the improved AlexNe + SENet. **(F)** is our ASCE.

In order to evaluate the performance stability of different model configurations in wheat leaf disease identification, more accurate confidence intervals and statistical tests are provided. We used the interval graph to show the accuracy distribution of six model variants tested independently on four diseases, as shown in **Figure 13**. Each scatter point corresponds to a test result, and the vertical aggregation degree and distribution range of scatter points directly reflect the fluctuation level of classification performance of each model.

**Figure 13** reveals that classical AlexNet yields a loose scatter with prominent low-value outliers, indicating insufficient test stability. Directly stacking CBAM and SENet onto classical AlexNet only marginally tightens the distribution, suggesting that the unoptimized shallow architecture cannot fully exploit the attention mechanisms. After structural optimisation, improved AlexNet concentrates the scatter points more effectively. Individually adding CBAM further narrows the spread, while adding SENet shifts the distribution upward, particularly for powdery mildew and leaf rust. The full ACSE model produces the most compact scatter across all four diseases, with accuracy concentrated in a high-value range and low outliers largely disappearing. This progressive convergence statistically confirms that structural optimisation, together with the synergistic integration of dual attention mechanisms, enhances classification robustness and test stability.

**Figure 14:**
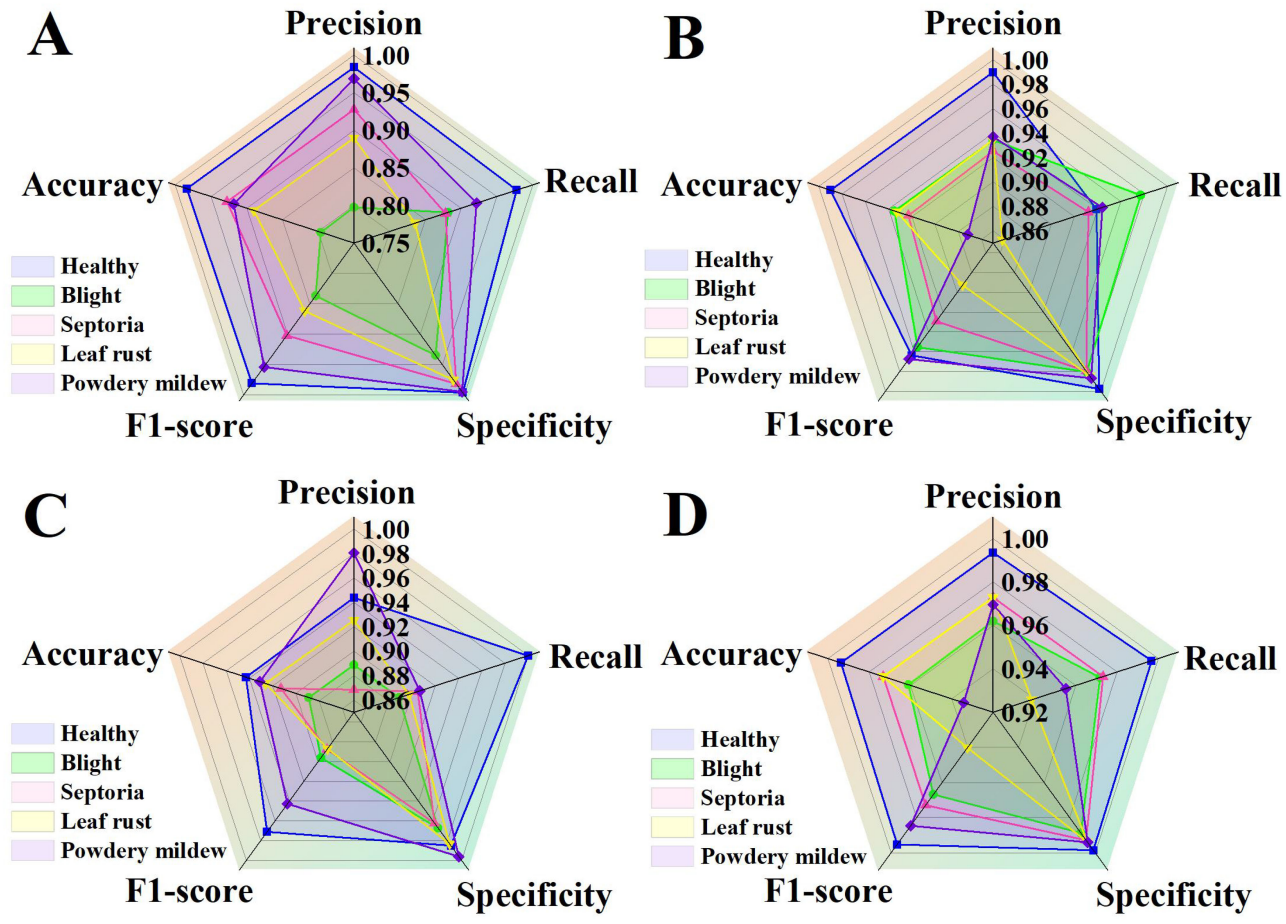
Comparisons of evaluation indicators of different models. **(A–D)** show radar chart results for five evaluation metrics of MobileNet V2, EfficientNet V2, Swin Transformer, and the proposed ACSE, respectively.

Radar charts facilitated multi-dimensional performance comparisons for wheat leaf disease classification by visualizing multiple evaluation metrics concurrently in polar coordinates. They integrated multi-index data and provided optimization guidance through spatial characteristics, with results depicted in **Figure 14**.

According to the quantitative analysis results in **Figure 14**, the detection model proposed in this paper is significantly superior to three other types of models, such as MobileNetV2 and EfficientNetV2, in five key performance indicators. In particular, it is worth noting that the indicators of the comparative model are generally above 80%, while all indicators of the new model are more than 92% and show a highly balanced performance distribution in different dimensions, without obvious bias. This result shows that the model does not fit specific diseases and shows excellent generalization ability. In the current task of wheat leaf health status recognition, the robust diagnosis ability of the model provides a reliable technical basis for building an intelligent early warning system of plant diseases that covers multiple diseases and avoids misjudgment bias.

**Figure 15:**
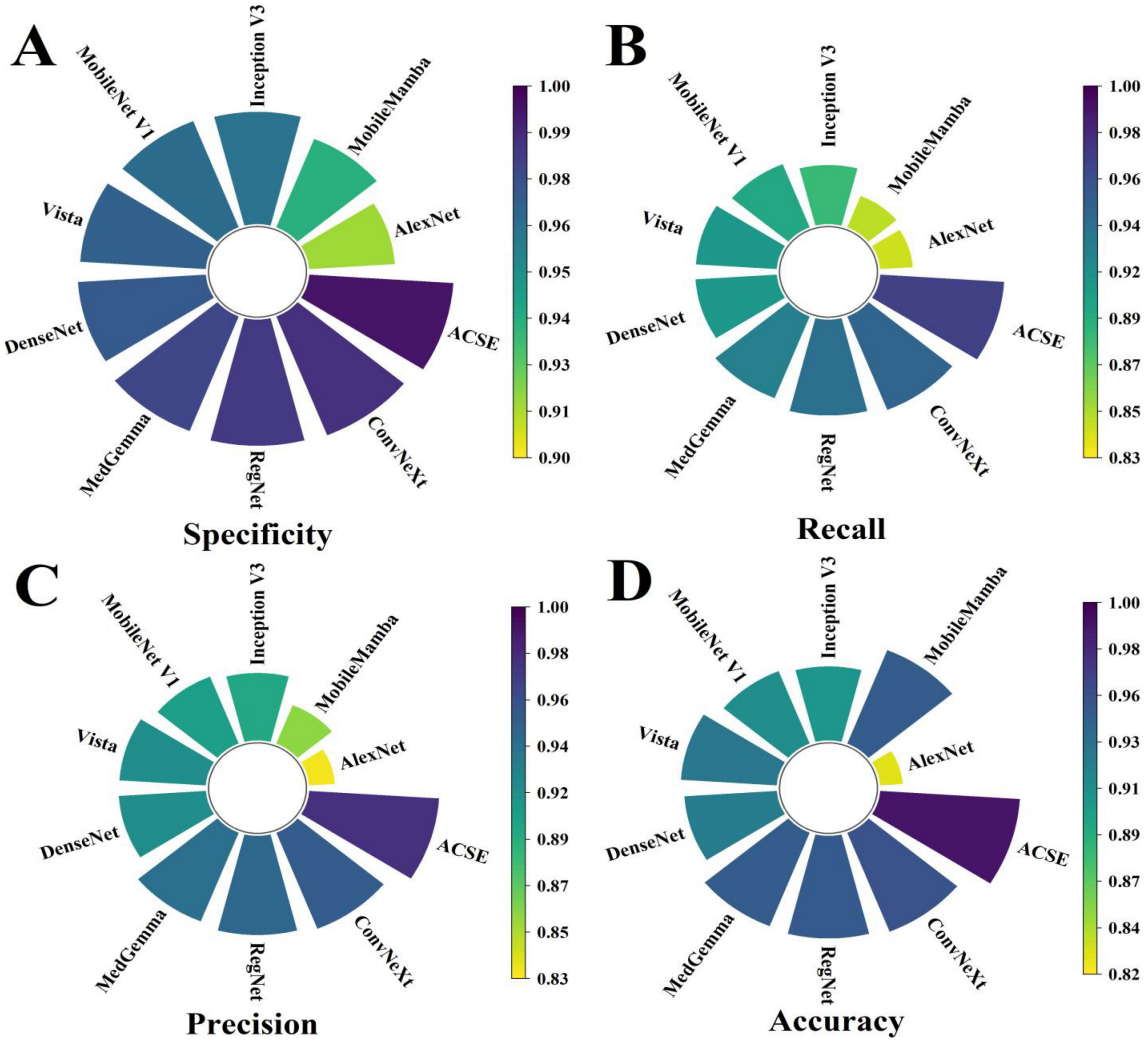
Comparisons of performance indicators of different models. Different sectors represent different models, and the radius length of each sector reflects the level of the corresponding performance indicator. **(a)** Specificity. **(b)** Recall. **(c)** Precision. **(d)** Accuracy.

To further evaluate the comprehensive performance of the proposed model, we compared our ACSE with several state-of-the-art deep learning models on specificity, recall, precision, and accuracy. The results are shown in **Figure 15**.

**Figure 15** employed a radial bar graph for visual model comparison. Subfigure (a) assessed specificity, demonstrating the ACSE’s superiority over AlexNet and MobileMamba. Subfigures (b) and (c) evaluated recall and precision, respectively; Vista and ConvNeXt exhibited strong positive class recognition, while our ACSE achieved optimal performance with minimal false positives. Subfigure (d) measured accuracy, confirming our ACSE as the most representative model with 98% accuracy. Overall, our ACSE surpassed mainstream models in core dimensions, including detection accuracy, coverage integrity, and robustness, supported by visual evidence.

**Figure 16:**
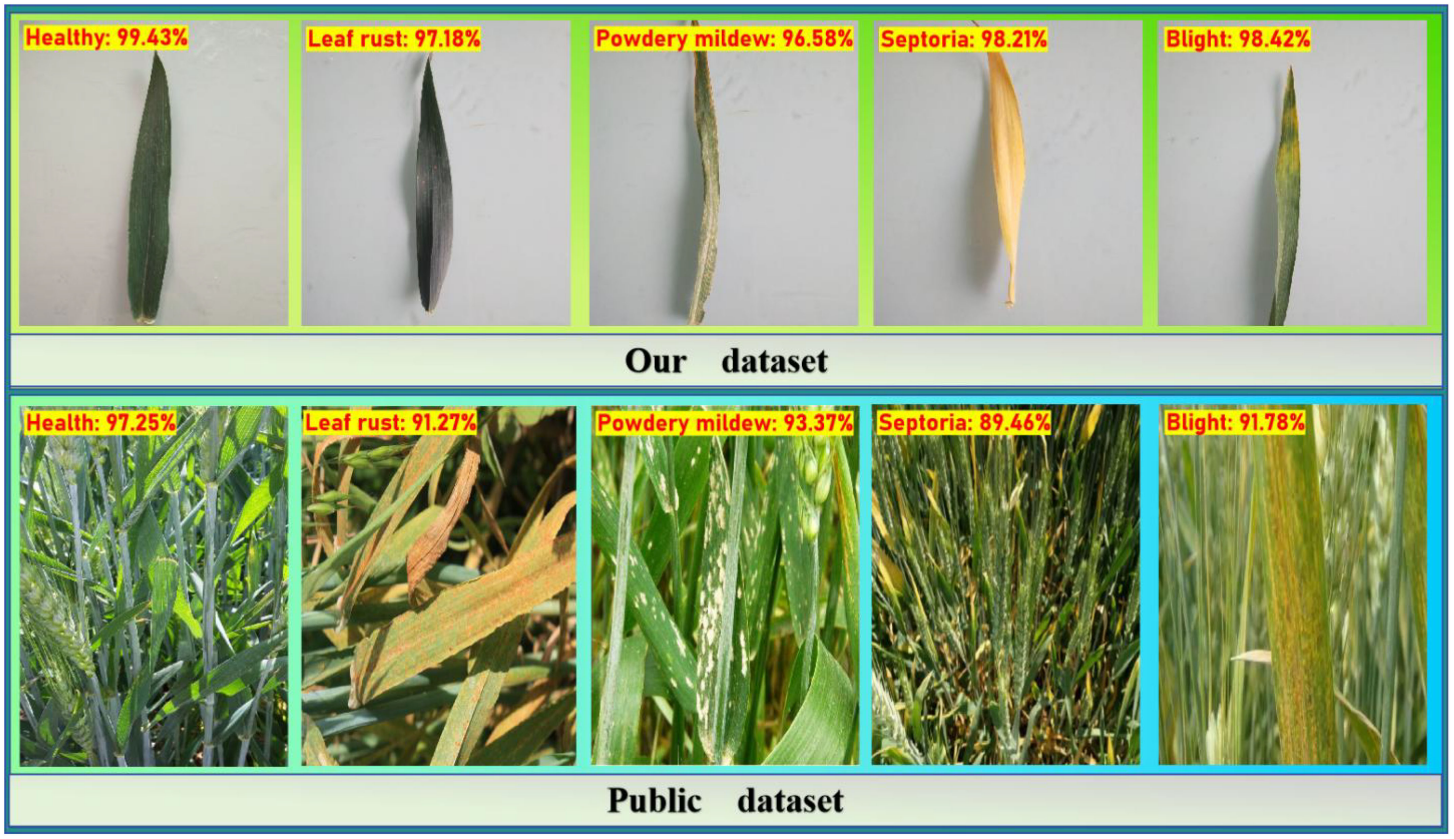
Predicted results on our and the open-access datasets.

We randomly selected some samples from the test set for testing. The prediction results are provided in **Figure 16**, and the recognition accuracy is above 89%.

**Figure 17:**
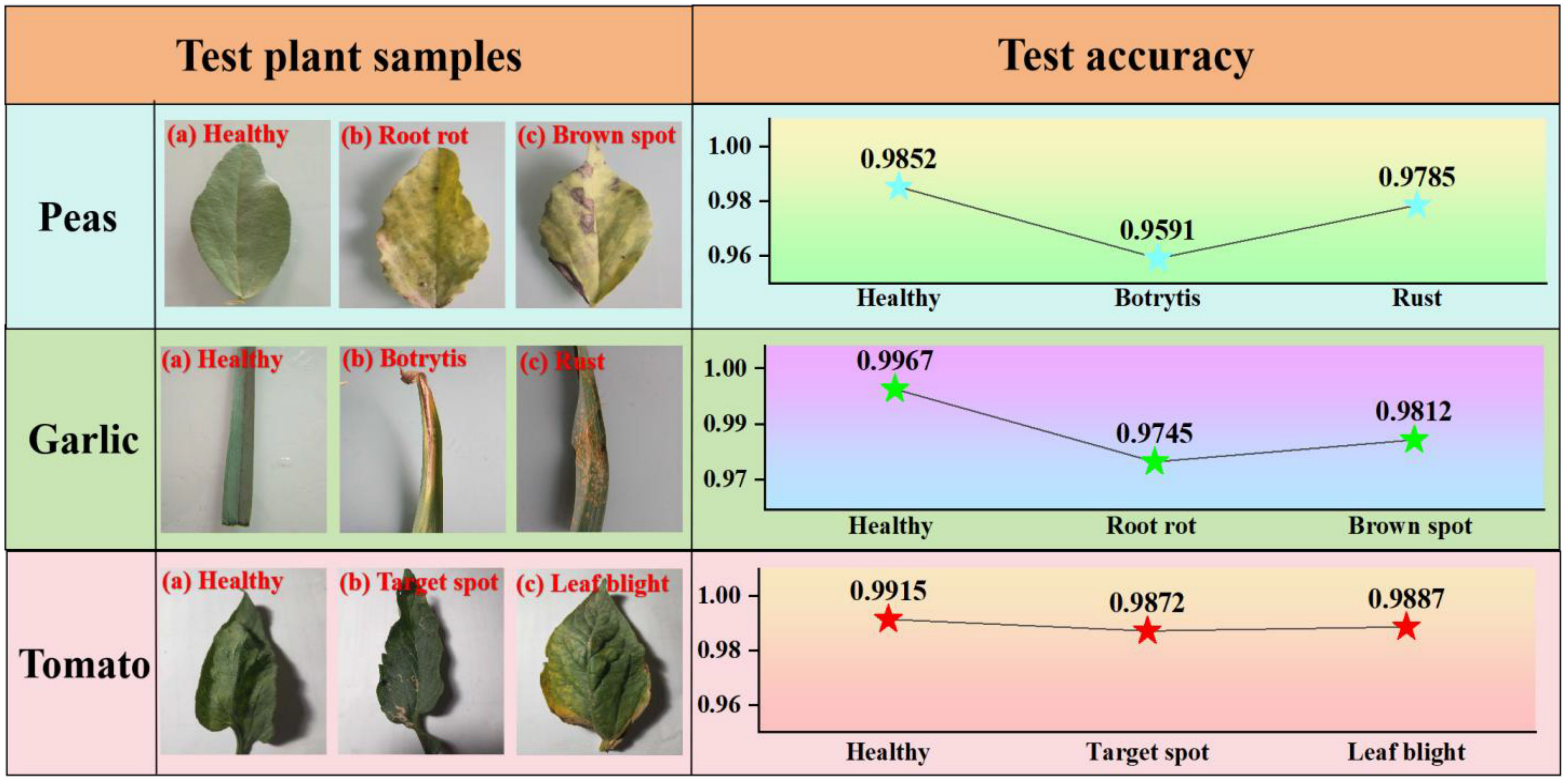
General performance of the proposed model.

Addressing the limited generalization of existing models across environments and species, our method demonstrated enhanced versatility and robustness. Validation employed a cross-species dataset (e.g., tomatoes, peas, garlic) simulating complex agricultural scenarios and unknown crop categories, with results in **Figure 17**. Based on the wide planting of peas, garlic, and tomatoes in agricultural production, this study selected the above three crops to construct the experimental data set. By integrating health images and two kinds of disease images, the complexity of the actual agricultural environment was simulated. The above cross-species experiments follow the same process as the wheat experiment: collect the field images of pea, garlic, and tomato, respectively; divide the training set, validation set, and test set, respectively; and use the ACSE model to complete the training and evaluation independently on the data of each crop. The experimental results show that the proposed method achieves 99.67% recognition accuracy on healthy pea samples. In the identification of garlic diseases, the identification accuracy of Botrytis disease was the lowest, 95.91%. In the identification of various tomato diseases, the accuracy rate remained above 98%. The above results show that the method has excellent generalization ability and robustness in the face of different species. It shows that the proposed model architecture has good adaptability to different crop disease data and can learn the discrimination characteristics of various diseases.

In addition, we collected the literature on wheat leaf diseases in the recent three years. The results are illustrated in **Table 6**.

**Table 6:** Comparisons of the proposed method with other literature from the past two years.

Authors., ref., year	Methods	Dataset size	Number of classes	Accuracy
Nigam et al. (2023)	Improved EfficientNet	10000	4	94.5%
Shi et al. (2023)	FFDNN	600	3	92.12%
Wen et al. (2024)	MnasNet-SimAN	7000	6	95.14%
Zhou et al. (2024)	REM-ShuffleNetV2	2238	2	96.72%
Chang et al. (2024)	ViT	4087	6	98.3%
Jiang et al. (2024)	Improved Yolov5s	15722	7	82.80%
Riccardo et al. (2024)	GranoScan	67302	6	96.00%
Li et al. (2025)	QY-SE-MResNet34	4248	6	89%
Xie et al. (2025)	MFFSNet	15430	4	97.38%
Proposed	ACSE	9390	5	98.51%

As can be seen in **Table 6**, a systematic review of wheat leaf disease identification studies over three years revealed deep learning models’ high performance, with accuracy consistently elevated. Variations occurred in dataset size, categories, and metrics: Chang et al. (2024) achieved 98.3% accuracy using ViT for six diseases, while Jiang et al. (2024) attained 82.80% with improved YOLOv5s across more categories. The ACSE model achieved superior accuracy (98.51%) on 9390 images of five diseases, excelling in multi-category classification while balancing efficiency and generalization.

## 5 Discussion

In recent years, deep learning technology has significantly promoted the development of plant leaf disease classification research and provided key technical support for intelligent monitoring of agricultural diseases. With the wide application of the Internet of things, the field is evolving towards the direction of high precision and lightweight coordination. However, the recognition ability of the existing models in the complex field environment is still insufficient. From the confusion matrix in **Figure 9D**, it can be seen that the misclassification of our ACSE mainly occurs in the early stage of the disease. For example, a small number of Septoria leaf blotch samples were misclassified as leaf blight, and a small number of powdery mildews were misclassified as Septoria leaf blotch. Looking back on these wrong samples, we can see that at this time, the disease spots have not been fully developed, and the leaf surface only shows slight discoloration or sparse powder, and the early symptoms of different diseases are highly similar visually. In addition, water drops or dust spots on the blade surface will also cause local texture interference, which further increases the difficulty of discrimination. This phenomenon shows that it is difficult to reliably distinguish the early disease with blurred symptom boundaries by relying solely on the spatial texture and colour information of the RGB image.

At the methodological level, previous studies on wheat disease recognition mainly focused on the local feature optimisation of convolution architecture, and the attention mechanism was mostly introduced in the form of a single module. Our ACSE architecture proposed in this study embeds Senet and CBAM in the optimised AlexNet in a collaborative manner. Senet first performs feature filtering of channel dimension, and CBAM then introduces spatial attention based on channel attention, forming a gradual feature enhancement from channel filtering to spatial focus. The results of ablation experiments show that compared with the use of CBAM or Senet alone, the collaborative fusion of CBAM and Senet shows an obvious complementary enhancement effect in accuracy and generalisation ability. Compared with the 98.3% accuracy rate achieved by Chang et al. (2024) based on VIT, the parameters of our ACSE under similar accuracy are much smaller than those of the other party, and the feasibility of edge deployment is higher. Compared with mffsnet (97.38% accuracy, four classification tasks) proposed by Xie et al. (2025), our ACSE has achieved higher accuracy under the five classification setting. Saleem et al. (2025) and Usman et al. (2025) have shown that VIT and its variants have outstanding advantages in global feature modelling, but the CNN model performs better in the task of crop disease recognition with local details as the key to discrimination. This is consistent with the conclusion of this study, which further confirms that the improved CNN is more suitable for local detail-oriented tasks such as wheat leaf diseases.

However, there are still some limitations in this study. Under extreme field conditions such as strong light overexposure or severe blade occlusion, the recognition robustness of the model still has room to improve, and the current data set does not cover these complex scenarios adequately. At present, the input mode is only limited to RGB images, and the disease physiological information carried by multispectral or hyperspectral data is not fused, which limits further improvement of the recognition accuracy of early and subtle diseases. The existing framework only supports the identification of a single disease on a single leaf and cannot deal with the complex situation of multiple diseases coexisting on the same leaf. The changes in climate conditions, shooting angle, and disease severity will also affect the recognition accuracy. Several engineering obstacles need to be overcome to migrate our ACSE from an experimental environment to real field deployment. The current model relies on an independent GPU to complete reasoning. When deploying on edge devices with limited computing power, the model needs to be compressed by distillation and quantification. Whether the compressed accuracy can be maintained at an acceptable level remains to be verified. Field light fluctuation and shooting jitter will reduce image quality and reasoning reliability, so it is necessary to integrate a real-time mobile image enhancement module. Rathod et al. (2025) developed a smartphone crop disease detection system based on the lightweight DenseNet201 architecture, which achieved 96% accuracy on the enhanced PlantVillage dataset and supported real-time offline diagnosis for farmers, providing a reference technical route for the subsequent edge deployment of our ACSE. In addition, the acquisition of multi-crop and multi-disease data depends on the cooperation of agricultural institutions and expert annotation, which is costly. Moreover, The difference of disease phenotypes between different crops may lead to domain offset and weaken the performance of cross-crop recognition. The above problems pointed out a clear direction for the follow-up research.

## 6 Conclusions

In this work, our ACSE model achieves 98.51% classification accuracy across two datasets, demonstrating strong convergence. However, environmental complexities such as fog, rain, and snow cause partial image information loss during wheat leaf recognition, limiting performance in blurred scenes. Additionally, current models, including our ACSE, rely on single-device offline image recognition and fail to integrate multidisciplinary technologies like UAV aerial photography or multispectral sensors. This restricts models to single-mode, localized, offline processing, hindering real-time field monitoring and large-scale smart agriculture applications. To address these challenges, future work focuses on:

1. Collect multi-scene data sets with strong generalization: optimize data construction through the three-level strategy of multi-source collection, dynamic enhancement, and scene adaptation. The expanded samples cover different regions, farming patterns, and growth periods, and they supplement early and complex disease samples. Meteorological simulation, attitude transformation, and background fusion were used to simulate real-field interference. Combined with domain adaptive technology, cross-scene alignment samples are generated to reduce scene bias and improve model generalization ability.
2. Developing low-computation optimization schemes combining pruning, distillation, and quantization to minimize parameters and computations with controlled precision loss. Operator and memory scheduling will enhance edge hardware efficiency, while cloud-edge collaboration enables lightweight front-end feature extraction.
3. Building a multi-technology integrated monitoring system embedding lightweight models on UAV platforms for rapid disease localization. This system will fuse multimodal data (e.g., UAV imagery, spectral inputs) into a visual agricultural platform, enabling precision pesticide application and early warning. Continuous advancements in model compression and hardware acceleration will facilitate deployment of lightweight multimodal models on edge nodes, integrating image, spectral, and textual data for robust crop health monitoring and intelligent agricultural evolution.

With the continuous development of model compression and hardware acceleration technology, the lightweight multimodal large model is expected to be deployed on mobile terminals and edge nodes. Integrating images, spectra, and text, these systems overcome single-crop disease identification limitations, establishing comprehensive crop health monitoring to advance agricultural disease recognition toward intelligent, generalized, high-precision evolution.

## Data Availability

The original contributions presented in the study are included in the article/supplementary material. Further inquiries can be directed to the corresponding author.
